# Advantages, Factors, Obstacles, Potential Solutions, and Recent Advances of Fish Germ Cell Transplantation for Aquaculture—A Practical Review

**DOI:** 10.3390/ani12040423

**Published:** 2022-02-10

**Authors:** Jun Hyung Ryu, Lan Xu, Ten-Tsao Wong

**Affiliations:** Department of Marine Biotechnology, Institute of Marine and Environmental Technology, University of Maryland Baltimore County, Baltimore, MD 21202, USA; rjh@umbc.edu (J.H.R.); xulan1@umbc.edu (L.X.)

**Keywords:** aquaculture, fish, germ cell transplantation, germline stem cells, surrogate propagation

## Abstract

**Simple Summary:**

This review aims to provide practical information and viewpoints regarding fish germ cell transplantation for enhancing its commercial applications. We reviewed and summarized the data from more than 70 important studies and described the advantages, obstacles, recent advances, and future perspectives of fish germ cell transplantation. We concluded and proposed the critical factors for achieving better success and various options for germ cell transplantation with their pros and cons. Additionally, we discussed why this technology has not actively been utilized for commercial purposes, what barriers need to be overcome, and what potential solutions can advance its applications in aquaculture.

**Abstract:**

Germ cell transplantation technology enables surrogate offspring production in fish. This technology has been expected to mitigate reproductive barriers, such as long generation time, limited fecundity, and complex broodstock management, enhancing seed production and productivity in aquaculture. Many studies of germ cell transplantation in various fish species have been reported over a few decades. So far, surrogate offspring production has been achieved in many commercial species. In addition, the knowledge of fish germ cell biology and the related technologies that can enhance transplantation efficiency and productivity has been developed. Nevertheless, the commercial application of this technology still seems to lag behind, indicating that the established models are neither beneficial nor cost-effective enough to attract potential commercial users of this technology. Furthermore, there are existing bottlenecks in practical aspects such as impractical shortening of generation time, shortage of donor cells with limited resources, low efficiency, and unsuccessful surrogate offspring production in some fish species. These obstacles need to be overcome through further technology developments. Thus, we thoroughly reviewed the studies on fish germ cell transplantation reported to date, focusing on the practicality, and proposed potential solutions and future perspectives.

## 1. Fish Germ Cell Transplantation in Aquaculture

Germ cell transplantation is the technology for surrogate production of donor-derived gametes. Donor cells possessing the ability of self-renewal and differentiation into gametes are injected into recipients that can support the survival, proliferation, and differentiation of donor cells so that recipients can produce donor-derived gametes. This technology has been spotlighted as an alternative to resolve critical reproduction obstacles of commercially important fish species. Although surrogate gamete production through this technology has been achieved academically in various species, including salmonids, cyprinids, and others considered commercially important [1,2], no industrial applications have been reported indicating that it is not commonly used in aquaculture. It may result from that established transplantation models have fewer advantages than their actual reproduction process, or surrogate gamete production technology has not been mature and successful in the critical species that do need this technology to solve their reproductive problems. 

Thus, in this review, we discuss the advantages, important factors, obstacles, and recent advances regarding fish germ cell transplantation technology to understand its current status further and propose the solutions for its future commercial applications.

## 2. Germ Cell Transplantation Methods in Fish

Fish germ cell transplantation can be classified into four different approaches based on the type of donors and recipients, including blastula cell transplantation (BCT), primordial germ cell transplantation (PGT), gonadal germ cell transplantation into the peritoneal cavity of larvae (GPT), and gonadal germ cell transplantation into adult recipients (GAT). In the following sections, the principle, results, and other aspects of each method are presented.

### 2.1. Blastula Cell Transplantation (BCT)

BCT is carried out by transplanting blastula cells that possess pluripotency into recipient blastula embryos. Fish blastula embryo consists of about 1000 cells, including primordial germ cells (PGCs) at various numbers depending on species [3]. PGCs migrate and incorporate into gonads during embryonic development through Sdf1a/Cxcr4b signal pathway [4].

**Figure 1:**
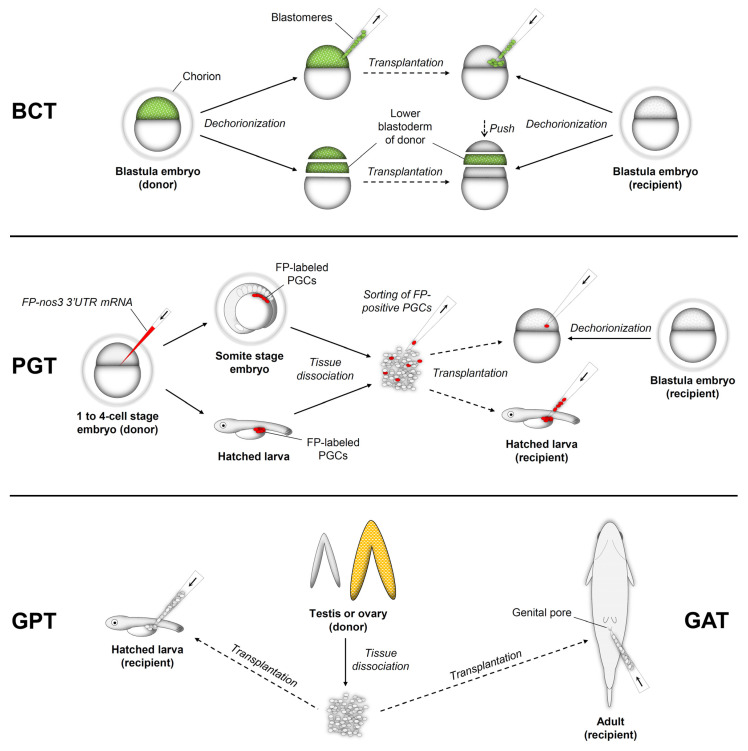
Methods of fish germ cell transplantation. BCT: blastula cell transplantation, FP: fluorescent protein, GAT: gonadal (testicular/ovarian) germ cell transplantation into adult recipients, GPT: gonadal (testicular/ovarian) germ cell transplantation into the peritoneal cavity of larvae, PGC: primordial germ cell, PGT: primordial germ cell transplantation.

Generally, less than 100 blastomeres or a lower part of the blastoderm are transplanted into each blastula embryo. Donor and recipient embryos are denuded by mechanical methods and/or treatments with proteases such as pronase or hatching enzyme to prepare donor cells or recipients [5,6,7]. When using dissociated blastomeres, donor cells are aspirated and transplanted into the blastoderm of blastula embryos with a fine needle [6,7]. In blastoderm transplantation, a lower part of the donor blastoderm is cut and placed onto the lower blastoderm part of a recipient embryo that has been removed half of the blastoderm, followed by pushing the upper half recipient blastoderm. Then, the donor and recipient blastoderms unite into one blastoderm [1,8] (Figure 1). The transplanted blastomeres or blastoderm integrate and mix with recipient cells. As the blastula cell population contains PGCs and somatic cells that can contribute to other lineages, chimerism can be observed throughout the organism that received blastula cells [9,10].

With BCT, the rates of germline chimera formation were variable and could reach 100% in some cases [10,11]. However, due to several manipulation steps such as removing chorion, low survival rates were noted in some recipients, such as rainbow trout (*Oncorhynchus mykiss*; 8.0%) [7] and zebrafish (*Danio rerio*; 26.1% or less) [6,8] (Appendix A, Table A1).

### 2.2. Primordial Germ Cell Transplantation (PGT)

PGCs are precursors of germline stem cells (GSCs) such as spermatogonia and oogonia. In fish, PGCs are specified by inheriting germplasm, maternally deposited in the oocytes, during early embryonic development [12]. In PGT, PGCs are generally harvested from embryos at somite stages by mechanical and enzymatic dissociation (Figure 1). When PGCs are labeled with fluorescence by microinjection of a fluorescent protein encoded mRNAs such as *DsRed/GFP-nos3* 3′ UTR [13], a fluorophore-conjugated dextran [14], or by transgenic fish expressing germ cell-specific fluorescent protein [15], they can be specifically collected under a fluorescence microscope [16], or by a fluorescence-activated cell sorting (FACS) [17,18]. For transplantation, purified PGCs are microinjected into the blastoderm of denuded blastula embryos [13] or the peritoneal cavity of anesthetized larvae [15]. Transplanted PGCs migrate and incorporate into developing recipient gonads, resulting in germline chimeras. Generally, 1–20 PGCs were transplanted into each recipient, and the germline chimera formation efficiencies were up to 89.5% [16] (Appendix A, Table A2).

### 2.3. Gonadal Germ Cell Transplantation into the Peritoneal Cavity of Larvae (GPT)

In addition to PGCs, larva recipients can also support the survival, proliferation, and differentiation of transplanted gonadal (testicular and ovarian) germ cells. Fish testes and ovaries carry GSCs called spermatogonia and oogonia, respectively, both of which possess the ability of self-renewal and differentiation into gametes. They can be harvested from donor testes or ovaries by mechanical and enzymatic dissociation. Like PGT, dissociated donor cells are microinjected into the peritoneal cavity of larvae (Figure 1). Of transplanted cells, spermatogonia and oogonia can colonize recipients’ gonads, forming germline chimeras.

Unlike PGT that used 1–20 PGCs for each recipient, more testicular or ovarian germ cells (up to 50,000 cells) were transplanted into each recipient (Appendix A, Table A3). The necessity of transplanting many cells may result from the relatively low proportion of GSCs in testicular or ovarian cell populations. For example, in immature rainbow trout (younger than 12 months old), the testicular and ovarian germ cells being *vasa*-positive accounted for less than 35.9% [19,20] and 11.9% [21] of total testicular and ovarian cell populations, respectively. Of the *vasa*-positive cells, only a small population can populate recipients’ gonads after transplantation. The cells expressing *dead end* (*dnd*) [22], *nanos2* [23,24] and/or *ly75* [25] have been considered to possess transplantability. The proportions of GSCs in the gonads may fluctuate according to the reproductive cycle and age of fish [22,26]. In the testes of immature fish such as 12 months old or younger rainbow trout [19] and 3 months old blue drum (*Nibea mitsukurii*) [27], all the germ cells were found to be spermatogonia. Therefore, immature fish have been preferred as donors due to the abundance of GSCs in their gonads (Appendix A, Table A3). Meanwhile, the rates of germline chimera formation were higher in testicular germ cell transplantation (up to 100%) rather than ovarian germ cell transplantation (34.5% or less; Appendix A, Table A3), which may be explained by the different proportions of GSCs between testicular and ovarian cells. On the other hand, regardless of the donor’s sex, both fish testicular and ovarian germ cells can contribute to either male or female germ lineages depending on the sex of recipients [21,28,29], indicating that testicular and ovarian germ cells possess the sexual plasticity and their fates are determined by recipients. Thus, both testicular and ovarian germ cells can be used to produce donor-derived sperm and eggs. This feature enables mono-sex seed production through germ cell transplantation technology (see Section 6.1).

### 2.4. Gonadal Germ Cell Transplantation into Adult Recipients (GAT)

Despite the mature immune system in adult recipients that can reject exogenous cells, transplanted gonadal (testicular and ovarian) germ cells can colonize adult recipients’ gonads and differentiate into functional gametes in fish (Appendix A, Table A4). It is thought that fish testis and ovary have the immune privilege provided by supporting cells such as Sertoli cells similar to mammalian testes [30]. Germ cells harvested from testes or ovaries with mechanical and enzymatic dissociation are transplanted into adult recipients by injecting cells directly into the gonads with a surgical incision or through the genital pore without incision (Figure 1). In general, a larger number of cells (2 × 10^6^–1 × 10^7^) were used for GAT since the gonad size of the adult recipients is significantly bigger than that of larva recipients (Appendix A, Table A4).

Since early embryos or larvae are used as recipients for BCT, PGT, and GPT, it takes a long time to obtain mature gametes through these technologies. Using adult recipients is an ideal solution to shorten the generation time of donor species because the recipients are already sexually mature. For example, in blue drum and Nile tilapia (*Oreochromis niloticus*), donor-derived sperm could be obtained at 7 and 9 weeks post-transplantation, respectively [31,32]. In addition, the survival rate of adult recipients was higher (greater than 75%) than those of other approaches. The rates of germline chimera formation in GAT were 5.0–33.3% (Appendix A, Table A4).

## 3. Advantages of Germ Cell Transplantation in Aquaculture

### 3.1. Shortening Generation Time

Selective breeding has been utilized to develop superior strains of commercial fish species that attain characteristics such as fancy appearance, improved growth performance, tolerance to environmental stressors, or disease resistance [33,34,35]. The processes require several generations to establish superior strains, which may take a long time depending on the target species. However, with germ cell transplantation using a recipient species with a shorter generation time, selective breeding can be achieved within a significantly reduced period. For example, in salmonids, Chinook salmon (*Oncorhynchus tshawytscha*) mature at 3 and 5 years in males and females, respectively [36], whereas rainbow trout reach sexual maturity at 1–2 years in males and 2–3 years in females [37]. By transplanting Chinook salmon’s germ cells into rainbow trout larvae, donor-derived sperm and eggs could be produced in rainbow trout recipients in 2 years [2,38]. Moreover, when this approach is applied to sturgeons with a much longer generation time, the breeding program might be significantly accelerated. Pšenička et al. [39] reported that GPT between Siberian sturgeon (*Acipenser baerii*; donors) and sterlet (*Acipenser ruthenus*; recipients) resulted in donor germ cells being incorporated into recipient gonads. Theoretically, if the recipient sterlet can produce Siberian sturgeon’s gametes, it is possible to obtain functional gametes of Siberian sturgeon 13–23 years earlier than the original sexual maturation timeline [39]. Furthermore, when germ cells are transplanted into the adult recipients (GAT), the generation time would be shortened more. For example, Nile tilapia testicular germ cells allotransplanted into adult males differentiated into functional sperm in 9 weeks, much shorter than the time usually needed to reach sexual maturity (around 6 months) [32].

### 3.2. Achieving Gamete Production of Semelparous Fish Recurrently through Multiple Seasons

Semelparous fish, such as most Pacific salmons, can only spawn once throughout their life cycle [40]. Although few populations of Chinook salmon survive after spawning and even repeat the spawning in the hatchery condition [41], most of them undergo deterioration and die soon following their first spawning season. Due to this characteristic, reusing the broodstock of these species reared for several years is impossible for the next round of seed production, meaning that farmers must repeatedly spend several years raising the new broodstock for only one-time use. This ineffectiveness can be mitigated by germ cell transplantation between semelparous fish (donors) and iteroparous fish (recipients). Indeed, semelparous Chinook salmon-derived sperm and eggs were produced by iteroparous recipient rainbow trout for multiple spawning seasons [2,38]. The surrogate production of semelparous germ cells in iteroparous recipients enables farmers to reduce the efforts and costs for broodstock management.

### 3.3. Solving Bottlenecks of Broodstock Maintenance

Effective broodstock maintenance and high-quality seed production are keys to successful aquaculture development. Of commercially important species, however, some fish are difficult or expensive to be domesticized to broodstocks that produce high-quality gametes. For example, Pacific bluefin tuna (*Thunnus orientalis*) and Southern bluefin tuna (*Thunnus maccoyii*) are highly popular and valuable marine fish that grow up to several hundred kilograms and require 3–5 years to reach sexual maturity [2]. Yet, due to their large body and long generation time, larger sea cages or land-based tanks for an extended period are needed for seed production of these species. In addition, as these fish are susceptible to light and noise, especially when they spawn, environmental conditions near the rearing facility should be controlled carefully [42,43]. All the above requirements translate into a considerable cost of maintaining the broodstocks. So far, only a few countries have achieved the reproduction of captive or reared bluefin tunas, despite their high demands and values [42,44], implying the difficulties in seed production of these species. To overcome these difficulties, xenotransplantation has been attempted with recipient species with advantages of a relatively smaller body, tolerance to environmental changes, shorter generation time, and well-established reproduction methodology, including blue drum [45], chub mackerel (*Scomber japonicus*) [22], Eastern little tuna (*Euthynnus affinis*) [46], yellowtail kingfish (*Seriola lalandi*) [47], or hybrid mackerel [48]. Although the successful production of gametes derived from the donor germ cells has not been reported, the incorporation of transplanted germ cells into recipients’ gonads was noted in these studies, indicating that these recipients could support the survival and migration of germ cells from bluefin tunas. Once mature bluefin tunas’ gametes are produced by one of these recipients, the vast rearing facility and rearing cost for an extended period would not be needed for seed production of bluefin tunas. The offspring would be produced cost-effectively with fewer risks, which can mitigate the limitation of seed availability and improve the productivity of bluefin tuna aquaculture. Besides bluefin tunas, this technology can also be applied to other fish species that encounter similar barriers described above.

### 3.4. Preservation and Restoration of Superior Strains Using Cryopreserved Cells

As the management of superior broodstocks developed by breeding programs or other approaches is very important for stable and high-quality seed productions, gamete preservation is highly recommended to prevent accidental losses of these valuable broodstocks [49]. Although the restoration of strains using cryopreserved sperm is possible by breeding through a few generations, it is only applicable to fish species that follow the male heterogamety sex-determination system (XX/XY), in which both X and Y chromosomes can be recovered from sperm. In cases of fish that have a female heterogamety sex-determination system (ZZ/ZW), the female’s genetic resource, the W chromosome, cannot be preserved by sperm (Z chromosome only) cryopreservation. While cryopreservation is successful in most fish sperm, it has not been achieved in fish eggs or embryos due to their low permeability, large volume, and yolk mass [50]. Unlike eggs or embryos, blastomeres [9,51], PGCs [52,53], spermatogonia [54,55], and oogonia [28,56] that can contribute to germ lineages have successfully been cryopreserved. Moreover, after transplanting these cryopreserved cells, the productions of donor-derived gametes have been achieved in many fish species [57]. Thus, the complete preservation and restoration of fish strains can be accomplished by cryopreservation and transplantation of blastomeres, PGCs, or ovarian germ cells, combined with sperm cryopreservation. This approach is imperative to preserve the valuable strains of several commercial fish that may have a ZZ/ZW sex-determination system, including flatfish [58], eels [59], and tilapias [60].

Notably, the production of germline chimeras was feasible by transplanting germ cells harvested from rainbow trout that were frozen with entire fish and kept in a deep-freezer (–80 °C) without using cryoprotectants. The viable germ cells could be harvested from frozen individuals of various sizes (18.8–203.9 g) [61]. These results imply that the preservation and restoration of fish lines can be achieved more practically even when standard cryopreservation methods are not available.

## 4. Factors Affecting the Success of Germ Cell Transplantation

Appendix A, Table A1, Table A2, Table A3 and Table A4 show that the transplantation success rates are highly variable among different studies. It is difficult to directly compare results from each study since most studies were conducted with various species and conditions by different researchers. Nevertheless, there are some factors noteworthy for achieving successful transplantation.

### 4.1. The Number of Donor Cells

The number of cells transplanted appears to have positive correlations with the success rates of transplanting testicular cells. In medaka (*Oryzias latipes*), larvae transplanted with 3000 or more unsorted testicular cells showed significantly higher colonization rates than those of recipients that received less than 3000 cells [55]. In rainbow trout and blue drum, similar patterns were observed, too. When 3000 or fewer unsorted testicular cells were transplanted into larvae, the colonization efficiency was in the range of 1.1–29.0% [19,20,62]. It increased up to 63.3% by transplanting more than 3000 testicular cells [63] (Appendix A, Table A3). While no comparative studies on the effect of donor cell number on colonization efficiency have been reported among adult recipients, transplanting 2 × 10^6^–1 × 10^7^ testicular or ovarian cells resulted in 37.5–100% colonization (Appendix A, Table A4).

The number of transplanted PGCs also affects the gonadal colonization efficiency in recipients. Li et al. [64] reported that when Dnd-overexpressed blastula embryos that contain more PGCs (~2.5 fold) employed as donors, higher germline chimerism after blastomere transplantation could be achieved, compared to regular blastula donors (81.2% vs. 47.2%). On the other hand, many donor cells do not seem to be necessary when transplanting blastomeres or PGCs. For example, 20–100 blastomeres were enough to generate germline chimeras in medaka [64], rainbow trout [7], and zebrafish [6]. For PGCs, germline chimeric goldfish and zebrafish could be obtained by transplanting a single PGC into blastula embryos [16,17,65]. In salmonids, 5–20 PGCs transplanted into larvae could colonize recipient gonads and undergo differentiation into functional gametes [15,66].

### 4.2. Purity of Donor Cells (Enrichment)

The purity of GSCs in donor cell populations can also affect the success rates of germ cell transplantation. Among the cells that constitute gonads, including germ cells at various stages, supporting cells, fibroblasts, and red blood cells, only GSCs, namely spermatogonia and oogonia, have the ability to colonize, self-renew, and differentiate into functional gametes in recipient gonads [21,67]. Thus, when an equal number of cells is transplanted, a higher success rate is expected if the enriched cells rather than crude cells are used. Several approaches have been attempted for the enrichment of GSCs, including Percoll density gradient centrifugation (PDGC), differential plating (DP), centrifugal elutriation (CE), FACS, magnetic-activated cell sorting (MACS) (Appendix A, Table A2, Table A3 and Table A4). In the following sections, the principle, enrichment effects, advantages, disadvantages, and applications of enrichment methods are presented.

#### 4.2.1. Percoll Density Gradient Centrifugation (PDGC)

PDGC is a cell separating method based on cell density using Percoll solution that consists of colloidal silica particles [68]. The cell suspension from tissues is loaded onto the top of layered Percoll solutions with different densities. After centrifugation, desired cells harboring a specific range of densities, which form bands between Percoll layers, are harvested for transplantation. For example, in zebrafish and medaka, as GSCs were abundant in 25–35% density Percoll fractions, cells harvested from these fractions were subjected to transplantation [69,70].

PDGC is the most widely used method for fish GSCs enrichment as its procedure is quick and straightforward without the need for special techniques and equipment. However, high purity is not expected because it is challenging to separate GSCs from other cells having similar densities. In loach (*Misgurnus anguillicaudatus*), 30–36% density fraction consisted of about 60% of type A and early-type B spermatogonia [71]. In olive flounder (*Paralichthys olivaceus*), the proportion of *vasa*-positive ovarian cells increased from 37.8% to 83.6% after PDGC [72]. In Siberian sturgeon, cell populations containing *vasa*-positive testicular cells (79.4% of total) and ovarian cells (70.8% of total) were obtained by PDGC [39].

#### 4.2.2. Differential Plating (DP)

DP is the cell separation method utilizing different adhesive characteristics among cells. After short-term incubation of cells on the substrata with culture medium, adherent cells can be separated from non-adherent cells by gentle pipetting or agitation. In general, as GSCs attach to substrata weakly, they can be separated from somatic cells such as fibroblasts that show relatively tight attachments [73,74]. In rainbow trout, *vasa*-positive cells were obtained at higher than 90% purity from immature testes by a series of DPs [75,76].

Since DP is simple and does not need special materials or devices like PDGC, it has also been widely used for cell enrichment. However, cell separation by DP may cause contamination with the cells harboring a similar adhesiveness to GSCs. Moreover, as DP takes up to several days, which is relatively longer than other methods, it raises a potential risk of spontaneous cell differentiation during in vitro culture [77,78]. On the other hand, DP would be a great tool when GSC-specific selective adhesive molecules become available. For example, in mice (*Mus musculus*), laminin was used for positive selection of spermatogonia, which showed 3–4-fold increased colonization efficiency compared to unsorted testicular cells [79].

#### 4.2.3. Centrifugal Elutriation (CE)

CE enables researchers to separate cells based on their physical characteristics. Unlike other typical centrifugations, cells are exposed to an outward centrifugal force and an inward counter-flow in the CE separation chamber, which arranges cells according to their sedimentation velocity, depending on their sizes, shapes, and densities. Subsequently, cells are eluted in the collection chamber by adjusting outward centrifugal force and inward counter-flow [80]. Bellaiche et al. [23] obtained cell fractions containing higher than 90% purity of type A spermatogonia (ASG) from immature testes of rainbow trout by CE with pre-enrichment by PDGC.

CE requires special apparatus and well-developed adjustment conditions for effective enrichment [81]. As a result, it has only been applied to a few studies related to fish germ cell enrichment and transplantation.

#### 4.2.4. Fluorescence-Activated Cell Sorting (FACS)

FACS facilitates cell separations based on light-scattering parameters of cells. During sorting, cells are exposed to a laser in a liquid stream, and their fluorescent characteristics are measured. Then, an electrical charge (positive or negative) is imposed on an individual cell, which can be sorted by an electrostatic deflection system [82]. Thus, desired cells that have specific light-scattering properties can be isolated by this method [83]. Especially, cells harboring fluorescence can readily be enriched by FACS due to their distinguishable light-scattering property, such as strong fluorescent intensity. Labeling germ cells with fluorescence can be achieved by microinjection of a fluorescent protein encoded mRNAs [13] or a fluorophore-conjugated dextran [14,84], with fluorophore-conjugated antibodies, or by transgenic fish expressing germ cell-specific fluorescent protein [15].

With transgenic rainbow trout (p*vasa*-*GFP*) carrying germ cells expressing GFP, PGCs and ASG could be enriched to 93.2% [85] and 93.2% purity [19], respectively. In non-transgenic fish, *GFP*-*nos1* 3′ UTR mRNAs were injected into 1 to 4-cell stage embryos to label PGCs, which resulted in reaching 100% purity after FACS [17]. Fluorophore-conjugated antibodies that were raised against fish ASG surface antigens led to 70.7–80.9% ASG purity after FACS in brown trout (*Salmo trutta*) [86], Pacific bluefin tuna [45], and rainbow trout [20]. Without fluorescence labeling, the enrichment of ASG was achieved by only the parameters such as forward scatter (FS; for cell size) and side scatter (SS; for granularity). 75.6–94.9% ASG purity could be achieved in blue drum, Japanese char (*Salvelinus leucomaenis*), masu salmon (*Oncorhynchus masou*), and sterlet [19,87]. With the optimized Hoechst 33342 staining condition, ASG could also be enriched using a side population [88].

Generally, a high level of enrichment is expected in cell populations sorted by FACS [89], especially when transgenic fish expressing GSC-specific fluorescent reporters or GSC-specific antibodies are employed. Of these, transgenic fish that carry GSCs expressing fluorescent proteins may encounter restrictions for commercial purposes [90]. Thus, employing the antibodies against specific surface proteins of GSCs seems one of the appropriate options to obtain highly enriched GSCs for aquaculture applications. However, the purities of fish GSCs after FACS with antibodies were 70.7–80.9% [20,45,86], which are lower than the purities (75.6–94.9%) achieved in FS and SS methods [19,87] and the purities (higher than 90%) enriched by FACS with antibodies in mammalian studies [91,92,93]. In addition, rainbow trout GSC antibodies (No. 80 and No. 95) also labeled a small population of somatic cells [20], indicating that developing more specific antibodies for fish GSCs is required to achieve higher purity by FACS. On the other hand, even if the specificity of an individual antibody is not great, high purity could be achieved by combining multiple antibodies since it enables sorting an overlap of cell populations labeled by multiple antibodies and/or excluding cell populations labeled with antibodies specific to undesired cell populations. In mammals, 98.8–99.8% purity of spermatogonia could be achieved by FACS using three combined antibodies against EPCAM, CD49E, and HLA-ABC [93]. Additionally, FS and SS methods can be another choice since they can achieve 75.6–94.9% GSC enrichment in fish [19,87], although it may require optimizing sorting conditions for each species.

As discussed above, FACS with GSC antibodies would be helpful to obtain highly enriched GSC populations from non-transgenic fish, which will be appropriate for commercial applications. However, FACS requires expensive apparatus, special materials, and trained personnel. Moreover, FACS sorts cells individually, which is inefficient for larger samples due to its low throughput (10^7^ cells per hour) [62,89].

#### 4.2.5. Magnetic-Activated Cell Sorting (MACS)

MACS separates cells with magnetic columns and magnetic nanoparticles-conjugated antibodies against specific cell surface antigens. Cells bound to the antibodies magnetically attach to the column so that these cells can be separated from unbound cells [94]. When the highly specific antibody is employed, MACS facilitates obtaining highly enriched cell populations. In fish, the GSC enrichment by MACS was reported by one study in salmonids, which employed the No. 172 antibody, resulting in 68.6 or 81.7% of sorted testicular cells and 54.8% of sorted ovarian cells being *vasa*-positive, respectively [62]. Compared to mammalian studies that achieved purity higher than 95% by MACS [95,96], the purity of fish GSC was lower, implying that the No. 172 antibody was not very specific to GSCs. Indeed, this antibody labeled differentiated spermatogenic cells even though it was developed against ASG [62].

Different from FACS, MACS cannot separate cells with gates customized by light-scattering properties. Therefore, more specific antibodies will be required to achieve a higher GSC purity. Additionally, combining antibodies would also be worth trying to improve purity [97]. Unlike FACS, MACS require less expensive equipment and skill settings [98]. In addition, MACS is applicable to large scales as it requires less than 15 min to sort magnetically labeled cells, regardless of the number of cells [62]. Thus, if the effective MACS condition is established, it will be valuable for the enrichment and transplantation of fish GSC in aquaculture operations.

#### 4.2.6. Applications

As described above, each enrichment method has its advantages and disadvantages. The enrichment methods for fish GSCs are summarized in Table 1. Enrichment methods can be used solely or combinedly. By combining them, the limitation of each method can be mitigated, which would lead to higher purities than those of enrichment with a single method [70]. The improved purity of GSCs after enrichment resulted in an increase of colonization efficiencies (up to a 17.5-fold increase) compared to those of unsorted cells [19,20,62,70,73,86]. Improved colonization rates by enriching donor cells will contribute to efficient surrogate offspring production, reducing efforts for screening works. The highly effective enrichment method will be useful, especially when recipient fish or space is limited, but donor fish are abundant. By contrast, enrichment should be carried out cautiously when only limited donors are available since transplantable cells can partially be lost during enrichment [62,85].

For the applications in aquaculture, extremely enriched donor cells do not seem necessary because improved success rates of transplantation are also achievable by using the immature gonads that contain a higher percentage of GSCs (see Section 2.3) or increasing the number of donor cells (see Section 4.1). In addition, enough recipients carrying donor-derived gametes can be secured by increasing the total number of recipients. Moreover, CE, FACS, and MACS, generally considered options to achieve high purity, require expensive apparatus, well-trained personnel, and/or special materials such as specific antibodies, which might result in increased costs. Thus, the reasonable and balanced points between the high-level enrichment and the investment should be determined with attention to practicality.

Together, to improve the efficiency of germ cell transplantation effectively, the enrichment of donor cells will need to be conducted depending on the availability of equipment, materials, techniques, donor/recipient fish, and rearing capacity, considering its pros and cons.

### 4.3. Age of Recipients

Among the factors, the age of the recipients can significantly affect the success rate of germ cell transplantation. Seki et al. [55] reported a significant decrease in colonization rates when testicular donor cells were transplanted into larvae older than 11 days post-fertilization (dpf) compared to larvae at 7 or 11 dpf in medaka. Meanwhile, in rainbow trout, transplantation of PGCs into recipients at 35 dpf showed relatively higher success rates, whereas the transplantation at earlier stages (younger than 35 dpf) or later stages (older than 40 dpf) resulted in significantly lower incorporation rates of PGCs into the recipient gonads [15]. In this regard, Yazawa et al. [99] reported that the developmental stage of larvae recipients is critical for a successful germ cell transplantation. In the xenotransplantation study with blue drum and chub mackerel, a high success rate could be obtained when transplantation was carried out with the recipient larvae at the stages when their germ cells were not completely surrounded by the gonadal somatic cells. In terms of adult recipients, there have been no comparative studies on the transplantation efficiency among different ages of them.

On the other hand, using younger recipients may require additional manipulations, such as dechorionization, resulting in low survivals [15,55]. By contrast, high survival rates (greater than 75%) of recipients were observed in adult recipients (Appendix A, Table A4). Even though wild-type adults were treated with high temperature and busulfan for eliminating endogenous germ cells before transplantation, it did not cause significant decreases in the survival of recipients when treated with the optimized conditions [72,100].

### 4.4. Prevention of Endogenous Germ Cell Development in Recipient Gonads

Prevention of endogenous germ cell development can give rise to efficient donor-derived gamete production. The recipients harboring endogenous germ cells can result in a high proportion of undesired offspring, introducing additional efforts for screening the desired offspring. Indeed, the low and/or variable proportions of donor-derived offspring were seen when fertile recipients were used in blue drum (2.2–89.2%) [63], rainbow trout (0.1–40.5%) [37], and zebrafish (3.9–7.3%) [101]. Thus, recipients’ endogenous germ cell development should be suppressed for the efficient surrogate production of donor-derived gametes. To prevent endogenous germ cell development in the recipients’ gonads, interspecific hybridization, triploidization, knockdown/knockout of *dnd* gene, high temperature/busulfan co-treatment, irradiation, etc. technology has been applied (Appendix A, Table A1, Table A2, Table A3 and Table A4). In the following sections, the principle, effects, pros and cons, other aspects, and applications of each sterilization method, which has frequently been used, are introduced.

#### 4.4.1. Interspecific Hybridization

Interspecific hybridization has conventionally been utilized to induce sterility in fish by inhibiting normal meiosis through a mismatch of chromosomes [102]. Hybrid fish show various reproductive phenotypes, including unusual sex ratio, reduced fecundity, or sterility [48,69,103]. Some hybrid fish even do not carry germ cells in their gonads due to mitotic arrests of PGCs. No germ cells were detected in hybrids between male white croaker (*Pennahia argentata*) and female blue drum, eliminating the potential of endogenous gametogenesis [27]. Due to these characteristics, sterile hybrid fish have been utilized as recipients, achieving 100% of donor-derived offspring production [27,31,69,104].

Since interspecific hybridization does not require sophisticated techniques and special equipment, hybrids seem like a reasonable option for recipients, as long as their sterility and supporting capacity for donor germ cells are verified. The adult hybrid recipients that do not carry endogenous germ cells are considered the most effective option due to their sterility and a significantly shortened generation time after transplantation. With hybrids between male white croaker and female blue drum, 100% donor-derived gamete production could be achieved within 7 weeks after transplantation at the earliest [31]. Yet, due to the highly variable features regarding survival rates, gonadal development, and supporting capacity for donor germ cells among hybrids developed with the combination of distinct species and sex, preliminary studies need to be done before the employment. Currently, only a few hybrids that harbor germ cell-less gonads have been known [27]. Therefore, further investigations to find out germ cell-less hybrids will be required for the broader application to various donor species.

#### 4.4.2. Triploidization

Triploid fish also do not undergo normal gametogenesis due to a mismatch of chromosomes [102]. Triploids can be generated by blocking the extrusion of the second polar body or breeding diploids with tetraploids which were produced by blocking the first mitosis [105,106]. Various techniques such as temperature shock (heat/cold), high pressure, chemicals, and electroporation have been developed to produce triploid fish in multiple species [107,108,109]. Although some triploid fish can produce a small amount of milt or eggs, their gametes may not have fertility, or their offspring cannot develop normally [110,111]. Moreover, in some species, triploid fish do not produce sperm or eggs at all [28,54,112]. They, as recipients, successfully produced donor-derived sperm or eggs in several germ cell transplantation studies (Appendix A, Table A3).

In fish, 100% triploidization was reported in species including Atlantic salmon (*Salmo salar*) [108], channel catfish (*Ictalurus punctatus*) [113], and rainbow trout [114] generated by simple temperature shocks. However, 100% triploidy is not always guaranteed, even with the same species and conditions that achieved 100% triploidy in other studies [63,115]. Tetraploid fish that can produce triploids by breeding with diploids are only available in limited species, including rainbow trout and loach [116,117]. In addition, tetraploids can potentially produce haploid or aneuploid sperm. Even diploid sperm, which is desired, may have low fertilization rates due to their head being larger than the micropyle of haploid eggs [118,119]. These indicate that using tetraploid fish to prepare triploid recipients would not widely be applicable. Besides, triploid recipients have the potential to produce their own endogenous gametes in some species. Indeed, triploid male grass puffer (*Takifugu alboplumbeus*) recipients produced 37.5% of total offspring derived from endogenous germ cells [120]. Thus, triploid recipients do not appear to be appropriate for allogeneic transplantation in various species. It might be acceptable if triploid endogenous gamete production is at the minimum level, and donor-derived offspring can easily be distinguished from the individuals derived from endogenous germ cells of the recipients.

#### 4.4.3. Dnd-Knockdown

The *dnd* gene is essential for the migration and development of PGCs, and its role is conserved among fish [121]. Without an appropriate expression of Dnd, PGCs fail to migrate to the gonadal region, resulting in the formation of gonads without germ cells [122]. When utilizing antisense Morpholino oligomers-mediated Dnd-knockdown by microinjection [123,124] or immersion treatment using Vivo-conjugated Morpholino [125] with early-stage embryos, the sterility was induced efficiently. In several studies, these sterile recipients produced 100% donor-derived gametes after germ cell transplantation [123,124,126].

In most fish species, a single *dnd* Morpholino can successfully induce Dnd-knockdown, but multiple Morpholinos are needed when multiple *dnd* transcriptional variants generated by alternative splicing are present in the target species [127]. Moreover, as the embryo survival and knockdown efficiency can be significantly affected by the target sequence and concentration of Morpholino [127], identifying effective Morpholinos and optimizing treatment procedures are necessary for achieving high survival and sterility induction. Although Dnd-knockdown showed high sterilization efficiency, 100% sterility has not always been guaranteed [124,127]. Thus, screening desired embryos that do not carry germ cells in their gonads should be strictly conducted. This can be achieved by visualizing endogenous germ cells through labeling PGC via either transgenesis or co-injection of fluorescent protein-encoding mRNA stabilized by *nanos3* or *vasa* 3′ UTR [127].

#### 4.4.4. *Dnd*-Knockout

The development of the *dnd*-knockout line is another approach for producing sterile recipients. In zebrafish, *dnd*-knockout homozygous mutants were used as recipients. In the progeny test, the effectiveness of donor-derived offspring production was 100% in the sterile *dnd* mutant recipients and, in contrast, only 3.9–7.3% in the fertile wild-type recipients [101].

For generating *dnd*-knockout fish, the genetic information (*dnd* gene) and time (at least for two generations) are required to establish a *dnd*-knockout line. In addition, the screening of homozygous fish is necessary as only 25% of total offspring from heterozygous parents will be sterile homozygous fish. When the *dnd*-knockout line is developed based on transgenic fish expressing germ cell-specific fluorescent reporters, screening can be done efficiently by selecting fish that do not carry germ cells expressing fluorescent reporters. The drawbacks are the additional time needed to develop the transgenic lines and the regulatory hurdle of using them in a commercial-scale application [90].

#### 4.4.5. Co-Treatment of High Temperature and Busulfan

Co-treatment of high temperature and busulfan has been applied to adult recipients for ablating endogenous germ cells (Appendix A, Table A4). High temperature arrests meiosis and induces massive apoptosis of spermatogenic cells in fish [32,72,128]. Busulfan is an alkylating agent that produces intrastrand or interstrand crosslinks of DNA, blocking DNA replication, cell proliferation, and differentiation [129]. As such, busulfan has been used to deplete fish testicular germ cells [128]. In zebrafish, co-treatment of high temperature and busulfan showed a synergetic effect in turning 88% of testicular tubules into the states without germ cells [128]. After transplantation, the adult recipients treated with this method showed 1.2–52.2% offspring derived from donor cells of total offspring (Appendix A, Table A4). Although a comparison of the transplantation success between untreated recipients and germ cell-depleted recipients has not been reported in fish, germ cell depletion by the co-treatment is expected to increase the proportion of donor-derived gametes, which has been shown in mammalian studies that 80% of donor-derived offspring production was achieved [130].

The advantage of co-treatment with high temperature and busulfan is the applicability to wild-type adult fish. The recipients can be prepared in a few weeks with this method without any special techniques or equipment. However, with this treatment, endogenous germ cells were not completely ablated, and the remaining GSCs could recover and repopulate gonads [128], impeding successful germ cell transplantation. In addition, as an inappropriate treatment can cause high mortality and inefficient germ cell ablation [72], optimizing procedures to achieve successful endogenous germ cell depletion is necessary.

On the other hand, long-term treatments (longer than 45 days) at a high temperature (37 °C) induced permanent sterilization in Nile tilapia and Mozambique tilapia (*Oreochromis mossambicus*) [131,132,133]. However, in another study, a similar treatment failed to induce sterility in male Nile tilapia since they recovered their fertility after returning to the normal condition [134]. The tolerance to heat treatment likely varies depending on the species or even among different strains of the same species. Thus, further studies are needed to verify its applicability to various species and strains.

#### 4.4.6. Other Aspects

As described above, eliminating endogenous germ cells can effectively improve the efficiency of producing donor-derived offspring. Besides this, it has been considered that germ cell-ablated recipients would enhance the colonization of transplanted cells since less competition is anticipated between endogenous and transplanted germ cells. However, it is not conclusive that ablating endogenous germ cells always positively affects the colonization of donor cells in recipients. In some cases, the transplantation success rates were higher in recipient larvae injected with *dnd*-MO to ablate endogenous germ cells, compared to untreated control recipients or hybrid recipients between male pearl danio (*Danio albolineatus*) and female zebrafish [135,136]. Still, the frequency of recipients harboring donor germ cells was not significantly different between control and *dnd*-MO treated recipient larvae in puffers (40.0% vs. 40.5%) [127] and salmonids (63.2% vs. 70.0%) [124]. *dnd*-knockout zebrafish recipient larvae showed a colonization rate similar to that of wild-type recipients (5.0% vs. 6.7%) [101]. These might be caused by the unfitness of underdeveloped gonads that are germ cell-deficient. As discussed in Section 4.3, the age of recipients can affect the success rates of transplantation [15,99,137,138], and it has been known that the gonadal development of germ cell-deficient fish can be delayed compared to wild-type [8,139]. However, in those studies, recipients at the same age were used regardless of the recipient type, which probably created unexpectedly different gonadal environments, making the comparison between experimental groups difficult. Further studies will be needed to clarify the effects of ablating endogenous germ cells on the success rate of transplantation in the GPT model. The comparison study on the impacts of germ cell ablation on donor cell colonization in GAT approaches has not been reported yet. However, the positive impacts of ablating endogenous germ cells are expected, considering the higher colonization rate in treated recipients than untreated recipients in mice [140].

Another important aspect of sterile recipients is that some sterile fish have a male-biased sex ratio. For example, the germ cell-less hybrids between male white croaker and female blue drum have a phenotypical sex ratio of 98:2 (male:female) [27]. Moreover, all the germ cell-depleted zebrafish by Dnd-knockdown/knockout developed into males [101,141]. Germ cells appear to be a critical factor of sex-determination in some species [142,143]. Thus, when donor-derived eggs are required, the feminization treatment is needed for sterile recipients in which the male-biased sex ratio has been observed. Saito et al. [16] feminized Dnd-knockdown sterile zebrafish recipients with 17-β estradiol treatment to successfully obtain donor-derived eggs from 75.0% of treated recipients. Xu et al. [104] could obtain female adult recipients of hybrids between male white croaker and female blue drum by triploidization (26.3% female of total), which successfully produced donor-derived eggs.

#### 4.4.7. Applications

For the application of germ cell transplantation in aquaculture, reducing labor-intensive works, costs, and the preparation period are vital aspects. Thus, when selecting a sterilization method, the simplicity, sterilization efficiency, and required time will need to be considered beforehand. When the decision is made with careful considerations on the pros and cons of each method, purpose of transplantation, and availability of technologies, it will lead to a successful outcome such as 100% donor-derived offspring production in a shortened period with fewer efforts and costs. The summarized information of methods for sterile recipient preparation is presented in Table 2.

## 5. Obstacles and Potential Solutions of Germ Cell Transplantation in Aquaculture

### 5.1. Practical Acceleration of Selective Breeding Process

As described in Section 3.1, germ cell transplantation can reduce the time needed to obtain mature donor-derived gametes when recipients have a shorter generation time. Therefore, it would significantly accelerate the selective breeding process that requires multiple generations, especially for the species with a relatively long generation time, such as Chinook salmon and sturgeons.

On the contrary, xenotransplantations between species with a relatively short generation time do not seem to have that advantage. For instance, grass puffer was suggested as a recipient to reduce the generation time of tiger puffer (*Takifugu rubripes*). The expectation was that sperm and eggs of tiger puffer could be produced quickly by transplanting tiger puffer testicular cells into grass puffer larvae. However, donor germ cells were harvested from 1-year-old male tiger puffer, and the donor-derived sperm and eggs were obtained at 10 and 22 months post-transplantation, respectively [120,127,138]. Therefore, when using grass puffer larvae as recipients, it took 22 or 34 months to produce tiger puffer sperm or eggs, respectively. Given that 2-year-old male and 3-year-old female tiger puffer can reach sexual maturity [138], it is not convincing that grass puffer can make a significant difference in shortening tiger puffer reproduction. In salmonids, it was proposed that xenotransplantation using rainbow trout larvae recipients may shorten the generation time of Atlantic salmon [150]. Rainbow trout generally begin to produce sperm and eggs at the age of 2 and 3 years, respectively [150,151], which is only 1 year shorter than Atlantic salmon. In one study, donor sperm and eggs were produced 1 and 2 years post-transplantation with the donor cells harvested from 1-year-old male Atlantic salmon [150]. Similar to the xenotransplantation between tiger puffer and grass puffer, there was only minor improvement in shortening generation time. Thus, it is not anticipated that xenotransplantation can significantly reduce generation time in those species unless younger donors or matured recipients are employed.

Since selective breeding requires the phenotypic and/or genotypic information of each individual for selecting parents, it is very challenging to use donor cells harvested from the individuals at very early developmental stages that provide no phenotypic characteristics nor enough cells for transplantation. In addition, in the blastula transplantation approach, only 2.5% of total recipients produced donor-derived gametes in rainbow trout [7], indicating low efficiency. These imply a limitation of blastula transplantation in use for selective breeding. When it comes to larvae carrying PGCs, although genotyping is possible, it comes with a great risk of losing fish due to sampling procedures. As such, selecting donors by phenotypes is difficult, and the number of desired recipients would be very low. In salmonids, a rainbow trout larva at 30 dpf only carries 30–60 PGCs [152,153], which can be used only for limited recipient larvae that receive 5–20 PGCs per recipient [15,66,154]. Moreover, the germline transmission rates were lower than 20% (Appendix A, Table A2), which is not cost-effective, given the costs for genotyping and transplantation. Although pooled PGCs collected from dozens of larvae may enable securing more transplanted recipients, it is not suitable for the selective breeding program that requires maintaining the pure line of each broodstock strain.

Using adult recipients with relatively higher survival rates can be an alternative approach to practically reduce the generation time since donor-derived gametes could be produced much quicker after transplantation [31,32] (Appendix A, Table A4). In this approach, what matters is securing enough donor cells. The individuals that can provide enough donor cells can be subjected to the acceleration of selective breeding through germ cell transplantation. For example, an immature yellowtail (*Seriola quinqueradiata*) male individual (10 months old) could provide about 3.8 × 10^8^ testicular cells [155], which are enough at least for 30 adult recipients when 2 × 10^6^–1 × 10^7^ testicular cells are transplanted into each recipient. Once appropriate recipients are identified and available, this approach would be feasible. Moreover, unlike embryo or larva donors, grownup donors provide more phenotypic information for confirming their genotype, enabling a more accurate selection of the desired individuals. As such, the generation time would be shorter than larvae recipients, accelerating the selective breeding process.

### 5.2. Securing Enough Donor Cells for Transplantation via In Vivo or In Vitro Propagation

To obtain enough recipients with high success rates of transplantation, enough donor cells should be prepared to ensure that maximal cells can be transplanted into each recipient. As discussed in Section 4.1, transplanting more donor cells into larvae could result in higher colonization rates (Appendix A, Table A3). In particular, each adult recipient has generally been transplanted with 2 × 10^6^–1 × 10^7^ testicular or ovarian cells (Appendix A, Table A4), indicating that sufficient donor cells are necessary for this approach. However, the gonad size of the donors and its availability depends on species or other factors. Enough donor cells may not be obtained when suitable fish are rare and/or their gonads are too small, affecting the success rates of germ cell transplantation. As such, methods for germ cell propagation would provide solutions to increase the number of donor cells.

For in vivo germ cell propagation, the donor germ cells are transplanted into the recipient fish, which would provide an increased number of donor cells by proliferating in recipients. In zebrafish, the long-term maintenance and securing enough testicular germ cells could be achieved by subcutaneous transplantation of testicular tissue fragments or aggregates under the skin of recipient fish. The size of grafted testis fragments increased more than 20-fold over 3 months post-transplantation. Through a series of transplantations, the grafted tissues could be maintained for longer than 3 years, providing enough cells, including spermatogonia and viable sperm derived from donor cells [156]. This approach was also attempted in rainbow trout, and the functional sperm derived from donor cells was produced in autografted recipients within 5 months post-transplantation [157]. So far, this technique has been successful only when aggregates were made with the mixture of donor testicular cells and cells harvested from testes of inbred recipient strains [158], when immune-deficient recipients (*rag1* mutant) were employed [156], or when autografts were carried out [157]. The recipients allografted with donor testicular fragments showed no donor germ cells in where tissue fragments were transplanted after 9 weeks, indicating that donor cells were rejected by the host immune system [157]. Immunosuppressants such as tacrolimus and cyclosporine have failed to suppress the immune rejection toward allogeneic donor testicular cells [159]. Further studies are required for the successful protection of donor tissues from the host immune system since the grafted tissues were degraded even in the immunosuppressants treated recipients. On the other hand, in vivo propagation of donor cells may also be achieved by transplantation into recipients at an early developmental stage when the immune system is not mature yet [57]. When serial transplantations of limited donor cells into larvae that can support the survival and proliferation of donor germ cells are carried out, it would propagate and increase the desired donor germ cells. For this approach, the contamination with the recipient’s germ cells will need to be prevented by employing germ cell-deficient larvae such as germ cell-less hybrids or Dnd-knockdown/knockout fish.

In vitro propagation of germ cells has been attempted in various species. PGCs, spermatogonia, and oogonia could be maintained for several months in vitro, showing colony formation and proliferation. The cultured germ cells could also colonize and differentiate into gametes in the recipient’s gonads after transplantation [160]. However, only a few studies showed a significantly increased number of GSCs after culture. A 3-fold increase of spermatogonia was observed in zebrafish after 20 days of culture, using the medium with supplements including 2-mercaptoethanol, embryonic extract, mammalian growth factors, and serum [158]. Notably, in rainbow trout, a 37.8-fold increase in the number of cells was achieved by in vitro culture of spermatogonia together with rainbow trout Sertoli cell feeders in TS medium-3 for 28 days. During in vitro culture, the characteristics of spermatogonia were maintained, demonstrated by transplantation assay [147]. Considering that germ cells from other salmonids could be nursed in the microenvironmental conditions supplied by rainbow trout recipients after transplantation [2,19,62,86,150], this culture system is probably able to apply to other salmonids or extend to other fish to propagate donor germ cells. Further studies on enhancing proliferation, maintaining GSC characteristics, and prolonging the culture period without causing GSC differentiation would enable a sufficient supply of donor cells for germ cell transplantation.

Collectively, the in vivo or in vitro germ cell propagation will facilitate securing abundant donor cells for transplantation and obtaining more recipients successfully carrying donor-derived gametes, which would be beneficial for surrogate offspring production as well as the selective breeding with donors that can provide only limited donor cells.

### 5.3. Enhancing Transplantation Efficiency with Vulnerable Recipients during Early Development

Some fish species have low survival and high susceptibility to physical stresses during early development, which can cause low success rates after embryo manipulation and germ cell transplantation. Thus, considerable efforts will be required to obtain enough recipients carrying donor-derived gametes in these species. Given the circumstances, adult recipients can be an alternative option instead of larva recipients. Zhou et al. [148] reported that transplanted spermatogonial stem cells from olive flounder, summer flounder (*Paralichthys dentatus*), or turbot (*Scophthalmus maximus*) could colonize recipient gonads and differentiate into functional sperm when triploid olive flounder larvae were used as recipients. Even though at least 500 recipients were transplanted with donor germ cells of each species, only limited recipients (2.2 to 6.8%; estimated) carried donor-derived gametes at 2 to 3 years after transplantation due to their low survival during early development. By contrast, when allogeneic transplantation was conducted using adult male olive flounder co-treated with high temperature and busulfan, all recipients survived, and 11.7% of total recipients produced donor-derived functional sperms within 10 months after transplantation [72]. Because GAT had higher success and survival rates and a shorter generation time than GPT, GAT would be a superb option in these species and other species that encounter the same challenges of low survival rate during early development. Moreover, with the optimized transplantation conditions and appropriate sterile recipients, it is expected that enhanced efficiency and a higher proportion of donor-derived offspring would be achieved.

### 5.4. Choice of Compatible Recipient Species for Xenotransplantation

The appropriate recipients with extraordinary abilities to support the survival, proliferation, and maturation of donor cells and improve productivity by reducing rearing costs for broodstock management are critical for applying xenotransplantation in aquaculture. In salmonids, the frequently used recipient species are rainbow trout and masu salmon, in which sperm and eggs of Atlantic salmon, rainbow trout, or Chinook salmon were successfully produced [38,146,150]. Goldfish (*Carassius auratus*) are also considered to have the capability to produce gametes of Cyprinid donor fish [29].

Besides those successful cases, xenotransplantations have been attempted in many other species. Of those, testicular germ cells from Pacific bluefin tuna or Southern bluefin tuna were transplanted into the blue drum, chub mackerel, Eastern little tuna, yellowtail kingfish, or hybrid mackerel successfully colonized recipients’ gonads. In sturgeons, testicular or ovarian germ cells obtained from American peddle fish (*Polyodon spathula*), Chinese sturgeon (*Acipenser sinensis*), Dabry’s sturgeon (*Acipenser dabryanus*), or Siberian sturgeon were able to colonize gonads of Dabry’s sturgeon or sterlet recipients after transplantation. Yet, the production of donor-derived gametes has not been reported in those species (Appendix A, Table A3). Meanwhile, in xenotransplantations between fish with far phylogenetic distances (greater than 200 million years), PGCs from Japanese eel (*Anguilla japonica*) and sturgeon could be incorporated into zebrafish and goldfish gonads, respectively. However, the production of donor-derived gametes was not reported [161,162]. These results may imply a well-conserved PGC migration mechanism and pathway but incompatible gonadal microenvironments among fish, making it challenging in surrogate offspring production through xenotransplantation among the fish with a far phylogenetic distance.

On the other hand, germ cells from goldfish and loach could proliferate and differentiate into functional sperm in zebrafish recipients with more than 100 million years of phylogenetic distance (MYD) from donors. In contrast, the ovaries of recipients did not mature and produce eggs [1,16]. Productions of donor-derived sperm have been achieved by xenotransplantation within the same genus, family, or order [1]. The spermatogenesis of transplanted Jundia catfish (*Rhamdia quelen*; Order Siluriformes) germ cells was observed even in the gonads of Nile tilapia (Order Cichliformes) that has 229.9 MYD from Jundia catfish [1,163]. Different from male recipients, the evolutionary distance seems strongly associated with the supporting capacity of female recipients. The most distant species that produced donor eggs through xenotransplantation were goldfish and common carp (*Cyprinus carpio*), with only 34 MYD from each other [29]. Thus, the successful spermatogenesis of donor germ cells in male recipients does not guarantee functional oogenesis in female recipients of the same species, indicating that a more compatible ovarian microenvironment may be required for successful female xenotransplantation.

In addition, the property of eggs produced by xenogeneic recipients was more close to the egg property of the recipients rather than that of the donors. The size of pearl daino’s eggs produced by zebrafish recipients (705.6 μm) resembled that of zebrafish eggs (699.3 μm), while pearl danio showed the egg diameter of 815.9 μm [16]. In puffers, the eggs of tiger puffer produced by grass puffer had clear chorions and a diameter of 8.8 mm, which were similar to that of grass puffer’s eggs but much different from tiger puffer’s eggs that have milky chorions and a diameter of 12.6 mm [120,127]. More importantly, regardless of recipients (triploid or Dnd-knockdown treated), the eggs produced by most female grass puffer recipients showed poor hatching rates (lower than 25%), implying the challenges of maintaining egg quality in recipients’ ovaries. Considering that eggs produced by xenotransplantation between goldfish and common carp (34 MYD difference), and rainbow trout and masu salmon (14.2 MYD difference) showed comparable hatching rates with that of control [29,124], the low hatching rates from the xenotransplantation between tiger puffer and grass puffer (4.2 MYD difference) suggests that the egg quality produced by xenogeneic recipients is associated with maternal contribution process such as vitellogenesis and choriogenesis rather than the phylogenetic distance.

Thus, unless planning to use a verified combination of donors and recipients, the preliminary investigation of supporting capacity is necessary prior to selecting recipient species, especially when surrogate egg production is crucial. Meanwhile, body size, generation time, spawning frequency, susceptibility to stress, and other physiological and economic characteristics of recipients should also be considered to have benefits of germ cell transplantation.

## 6. Advanced Applications of Germ Cell Transplantation

### 6.1. Production of Mono-Sex Populations

Due to characteristic differences between male and female fish, the mono-sex population is beneficial for commercial aspects in various species. For example, the female seedlings of Atlantic salmon [164], olive flounder [165], and rainbow trout [166] are preferred in aquaculture as they either grow faster than males, or males generally reach sexual maturity earlier than females, resulting in the loss of growth potential and flesh quality [167]. In sturgeons, females are more valuable owing to caviar in their gonads. In contrast, some male-biased populations are favorable because of their growth performance or fancy appearances, such as Nile tilapia or ornamental fish [168,169]. Accordingly, diverse methods have been attempted to produce mono-sex populations in various species.

Rearing conditions, including high temperature, hypoxia, low pH, or stocking density, affected male or female-biased populations [170]. Manipulating rearing conditions is considered an ecologically friendly approach to producing mono-sex populations on a commercial-scale when environmental factors are critical for the sex-determination of fish. However, the sensitivity to environmental factors varies among fish species depending on their sex-determination mechanism. Regulating rearing conditions are less-effective for controlling the sex ratio for the species whose sex is mainly determined by genotype, such as rainbow trout [171]. For instance, in rainbow trout, high temperature affected sex ratio, but the treated populations were male- or female-biased depending on broodstock [172]. In addition, stressful conditions such as high temperature and high density compromise animal welfare [173].

Unlike environmental factors, the treatment of sex reversal chemicals, including sex steroid hormones, inhibitors, and antagonists, can effectively drive fish to male- or female-biased populations regardless of their sex-determination mechanism [174]. For instance, in rainbow trout, nearly 100% sex-reversed male population was obtained by treating 17α-methyltestosterone [175]. However, since increasing concerns associated with the adverse effects of sex steroids on the environment and humans, the regulations regarding sex steroids in aquaculture have become strict. For example, in the US, the use of 17α-methyltestosterone has been controlled by the Food and Drug Administration Investigational New Animal Drug (INAD) program (INAD #11-236 and #8557), which allows the use of this hormone only for limited species such as Nile tilapia, Atlantic salmon, and rainbow trout. Therefore, alternative approaches to producing a mono-sex population are needed for sustainable aquaculture.

Germ cell transplantation is one of the potential alternatives. By transplanting ovarian germ cells that carry XX chromosomes into sterile male recipients, milt containing only X sperm can be obtained and used to produce all-female fish [28,72]. On the other hand, one-quarter of YY males can be obtained from fertilizing normal X or Y sperm with X or Y eggs produced by female recipients that received testicular germ cells. Accordingly, all-male populations can be produced by mating YY males with XX females [176,177]. In fish that possess ZZ/ZW sex-determination system, the all-male or -female populations can be generated by conducting the above procedures reversely. In particular, when using sterile recipients (see Section 4.4), efficient production of 100% donor-derived all-male or -female offspring would be possible without using stressful rearing conditions or sex reversal chemicals, contributing to sustainable aquaculture.

### 6.2. Production of Sterile Fish through Transplantation of Germ Cells Carrying Mutated Somatic Genes

Reproductively sterile fish have advantages for aquaculture operations. First, sterility prevents sexual maturation that hinders growth and diminishes product value. Fish devote energy and deposit nutrition into gonads during sexual maturation, leading to ineffective feed conversion [178]. In addition, sexual maturation may hinder growth potential, resulting in smaller sizes [179,180] and ruin flesh quality and flavor [181,182]. Therefore, the prevention of sexual maturation is crucial in some commercial fish species, such as Arctic charr (*Salvelinus alpinus*) [183] and Atlantic salmon [180]. Second, genetic containment can be accomplished with sterile fish that prevent polluting wild populations’ genetic pool when selectively bred or even genetically modified farmed fish escape. Especially, sterilization is vital for containing transgenics and genome-edited mutants for their commercial applications [184,185]. Third, sterility is a means for producers to protect valuable strains from unauthorized propagation and unexpected technological leakages.

Sterility can be induced in various ways, including interspecific hybridization [27,69,186], triploidization [108,110,112,115], irradiation [187,188], *dnd*-knockout [101,189], or Dnd-knockdown by *dnd*-MO immersion [125]. Interspecific hybridization may not be practical when hybrids themselves are not commercially valuable. Additionally, hybrids’ reproductive phenotypes are highly variable depending on the combination of species/sex (see Section 4.4.1), limiting their applicability in aquaculture. For triploidization, the induction efficiency depends on species and methods. Triploid fish may produce functional gametes in some species (see Section 4.4.2), raising the risks of contaminating the genetic pool of the wild populations or unexpected leakages of valuable strains. Moreover, triploid fish may have low early survival, high deformity, and different behavioral and physiological characteristics from diploids [190], which might not be preferred in some cases. Although irradiation and *dnd*-MO immersion are useful methods to produce sterile diploids, avoiding undesired phenotypes of hybrids or triploids, the optimized condition is required to achieve 100% sterility [125,188]. Sterility can also be induced by breeding mutants such as *dnd*-knockout fish (see Section 4.4.4). Still, the limitation of this approach is that only 25% of sterile offspring can be obtained from heterozygous parents [101,189].

On the contrary, 100% sterile offspring can be obtained through transplantation technology in cases of sterility caused by mutated somatic genes essential for gonad development. In this approach, sterile wild-type recipients are employed to support mutant germ cell development. For example, sterile female homozygous *follicle-stimulating hormone receptor* (*fshr*) mutants could continuously be produced by germ cell transplantation in medaka. Donor cells carrying XX chromosomes were collected from homozygous *fshr* mutants and transplanted into sterile hybrid recipients. The resultant female recipients carrying mutant donor X eggs were mated with sex-reversed homozygous mutant fertile neo-male medaka, producing all-female sterile mutants [144]. As the mutation of *fshr* could induce sterility in other fish species [191], the *fshr* mutant model can potentially be useful for producing sterile fish in other species with male or female heterogamety sex-determination system. Besides the *fshr* knockout model, infertility was observed in male or female fish that harbor other mutated genes, including *gonadal soma-derived factor* [192], *estrogen receptors* [193], *luteinizing hormone* [194], or *luteinizing hormone receptor* [191], suggesting a broader potential of developing additional models for producing sterile fish. Together, germ cell transplantation with sterile wild-type or hybrid recipients would help the continuous production of homozygous sterile fish of these mutants for commercial purposes.

## 7. Future Perspectives

Since the first report of germline chimeric fish made by blastomere transplantation [6], many methods have been developed to generate germline chimeras in various species with diverse transplantation techniques. While germ cell transplantation technology has a promising potential to enhance aquaculture productivity and mitigate reproductive bottlenecks, its procedures involve many steps, including donor cell isolation, recipient preparation, and transplantation that require special apparatus, skills, and labor, which translate into substantial costs in its commercial applications. Therefore, its application needs to have unique and more significant benefits than the general breeding process can offer to gain cost-effective advantages. Notably, the commercially valuable species have apparent difficulties in broodstock management and/or seed production, such as bluefin tunas and sturgeons. Once the appropriate and effective recipients harboring significant benefits in reproducing desired donor species have been identified and established for each valuable species, the surrogate production model would be in demand by the aquaculture industry. Moreover, when combined with approaches for producing mono-sex populations or sterile fish, germ cell transplantation technology will provide a more efficient and ecologically friendly way to produce valuable seedlings. In particular, since the generation of 100% sterile mono-sex fish harboring mutated somatic genes is only achievable through germ cell transplantation, it is a unique opportunity to demonstrate this technology in commercial operations. Furthermore, with cryopreservation, transplantation technology would enable more practical and secure broodstock management.

To maximize the effectiveness of transplantation, adult recipients seem superior to embryo or larva recipients because it can significantly increase recipient survival rates and reduce the generation time of target species. Thus, as long as sufficient donor cells and sterile adult recipients possessing a supporting capacity for donor germ cells are available, adult recipients will need to be prioritized. Additionally, developing in vitro culture systems for germ cells would be one of the critical factors that can enhance the applicability of germ cell transplantation in adult recipients. In vitro propagation of germ cells will facilitate acquiring abundant donor cells, thereby obtaining enough recipients with higher transplantation success rates and reduced animal sacrifice. Mainly, this will be valuable for accelerating the selective breeding process with individuals carrying fewer germ cells.

**Figure 2:**
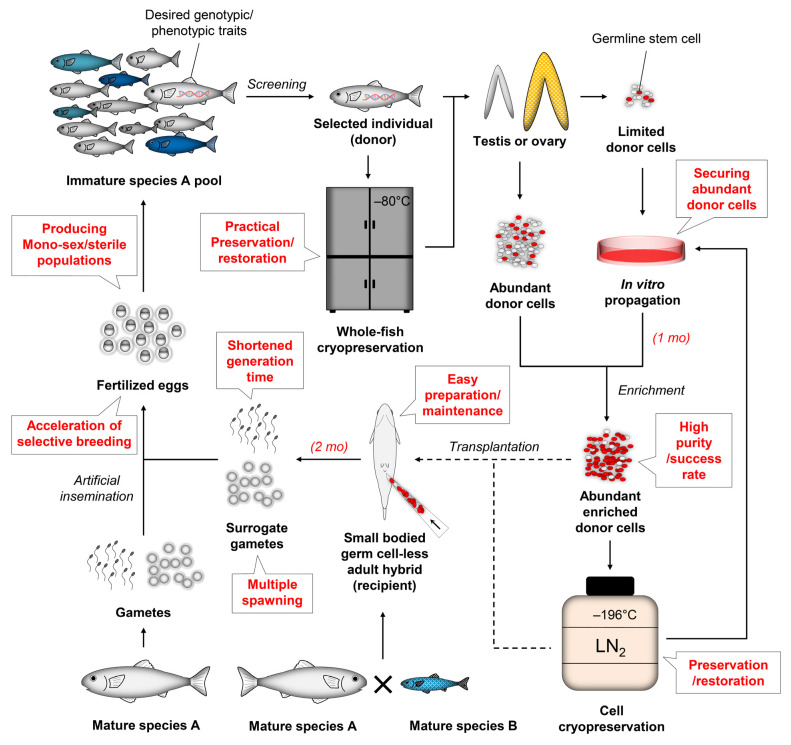
A suggested model to enhance the advantages and practicality of fish germ cell transplantation. Red bolds represent the benefits of the step or output. Red italics in brackets indicate the anticipated time for the step.

As discussed in Section 4, transplantation success rates are highly variable, and many factors are involved in accomplishing successful transplantation. Therefore, further studies for optimizing transplantation procedures will be required to achieve a stable and improved efficiency for aquaculture application. A suggested model to enhance the advantages and practicality of fish germ cell transplantation is illustrated in Figure 2. Taken together, germ cell transplantation technology can be utilized for various purposes in aquaculture. With suitable recipients, optimized transplantation conditions, and related advanced technologies, it will solve several reproductive problems and improve productivity across multiple valuable commercial species.

## Figures and Tables

**Table 1:** Enrichment methods used for fish germline stem cells.

Method	Principle	Advantage	Disadvantage	Enrichment of Fish GSCs
PDGC	Fractionating cells by their density with centrifugation	Simple procedure No need for special materials, techniques, and equipment	Low purity	The most frequently used method 60–83.6% purity [39,71,72]
DP	Positive or negative selection based on adherence of cells by in vitro culture	Simple procedure No need for special materials, techniques, and equipment	Low purity Relatively long procedure Potential risks of spontaneous differentiation during in vitro culture	Frequently used >90% purity by serial DPs [73,75] No available commercial molecules for positive selection
CE	Aligning and eluting cells by their physical characteristics with centrifugation	High purity expected Not requiring TGs or ABs	Requiring special sorting conditions and equipment	>90% purity by combining with PDGC [23]
FACS	Isolation of cells based on light-scattering properties	High purity expected Flexible sorting condition by customizing gates based on size, granularity, and fluorescent intensity of cells	Requiring special skills and equipment Requiring specific ABs against the surface protein of target cells, TGs carrying germ cells expressing FPs, or other specific sorting conditions Not suitable for large scales	Up to 100% purity of PGCs labeled with mRNA-*nanos3* 3′ UTR encoded FP [17] 93.2–99% purity with TGs [18,19,85] 70.7–80.9% purity using ABs [20,45,86] 75.6–94.9% purity without using TGs or ABs [19,87] Limitation of using TG fish for commercial application No available commercial fish ABs
MACS	Affinity based cell sorting with magnetic particles-conjugated ABs against cell surface proteins	High purity expected No need for special techniques or equipment Simpler than FACS Applicable to large scales	Requiring specific ABs against the surface protein of target cells	54.8–81.7% purity [62] No available commercial fish ABs

AB: antibody, CE: centrifugal elutriation, DP: differential plating, FACS: fluorescence-activated cell sorting, FP: fluorescence protein, GSC: germline stem cell, MACS: magnetic-activated cell sorting, PDGC: Percoll density gradient centrifugation, PGC: primordial germ cell, TG: transgenic.

**Table 2:** The sterilization methods used to prepare recipients for germ cell transplantation in fish.

Method	Principle	Advantage	Disadvantage	Recipient Case
Interspecific hybridization	Preventing normal meiosis by a chromosomal mismatch or arresting mitosis of PGCs	Simple preparation by breeding without any treatment Complete germ cell elimination (in germ cell-less hybrids)	Unpredictable reproductive phenotypes depending on species/sex combination	♂ *D. albolineatus* × ♀ *D. rerio* [69], *O. curvinotus* × *O. latipes* [144], ♂ *P. argentata* × ♀ *N. mitsukurii* [27,31,104]
Triploidization	Preventing normal meiosis by a chromosomal mismatch	Relatively simple procedure	Potential risks of endogenous germ cell-derived gamete production Unstable efficiency depending on species and method	*D. rerio* [145], *N. mitsukurii* [63], *O. masou* [28,146], *O. mykiss* [21,56,147], *O. latipes* [55], *P. olivaceus* [148], *T. alboplumbeus* [120,138]
Dnd-knockdown	Disrupting PGC development by MO-mediated inhibition of Dnd expression	High efficiency expected Possibility of complete germ cell elimination	Requiring special techniques, apparatus, or materials Not guaranteed 100% sterility	*C. auratus* [29,65], *D. rerio* [16,123,126], *O. masou* [124], *O. latipes* [64], *T. alboplumbeus* [127]
*Dnd*-knockout	Disrupting germ cell development by blocking Dnd expression through gene editing	Guaranteed 100% sterility in homozygous mutants Complete germ cell elimination	Requiring genetic knowledge, techniques, apparatus, and time to establish mutant lines Only 25% sterile fish of total	*D. rerio* [101], *O. mykiss* [38]
High temperature + busulfan co-treatment	Arresting meiosis and inducing apoptosis of germ cells	Preparable in a few weeks with wild-type adults	Incomplete germ cell ablation Requiring optimized conditions to achieve low mortality and successful germ cell ablation	*O. hatcheri* [100,149], *O. niloticus* [32], *P. olivaceus* [72]

Dnd: Dead end, MO: Morpholino oligomer, PGC: primordial germ cell.

## Data Availability

Not applicable.

## Appendix A

**Table A1:** Blastula cell transplantation in fish.

Donor			Recipient		% of Recipients with Gonadal Incorporation of Donor Cells	% of Recipients Producing Donor-Derived Gametes			Survival of Recipients (%)	% of Donor-Derived Offspring	Ref
Species	Fish	Cells	Species	Fish		Total	Male	Female			
*C. auratus*	All-female (XX)	A lower part of BD	Hybrid	♂ *C. carpio* × ♀ *C. auratus*, all-male	N/A	N/A	100 at SM	N/A	Con: 79.9 TP: 72.3 at 4 d	N/A	[11]
	WT	BMs	*C. auratus langsdorfii*	3N	3.1 at 4 d	N/A	N/A	N/A	Con: 65.8 CP + TP: 35.6 at 4 d	N/A	[9]
	WT	50–100 BMs	*D. rerio*	WT	7.3 at 24 h	N/A	N/A	N/A	73.3 at 24 h	N/A	[13]
*C. auratus langsdorfii*	3N	A lower part of BD	*C. auratus*	WT	N/A	N/A	N/A	100 at 3–4 y	Con: 91.4 TP: 98.4 at 3 d	3.1–89.3	[10]
*D. albolineatus*	WT	50–100 BMs	*D. rerio*	WT	6.6 at 24 h	N/A	N/A	N/A	96.8 at 24 h	N/A	[13]
*D. rerio*	WT or TG:*pRSV-LacZ* (*LacZ*/-)	20–100 BMs	*D. rerio*	Albino	N/A	17.9 at 2–3 mo	N/A	N/A	16.7 at SM	1.0–40.0	[6]
	WT	50–100 BMs	*D. rerio*	WT	9.0 at 24 h	N/A	N/A	N/A	86.7 at 24 h	N/A	[13]
	TG:p*vasa-DsRed2*-*vasa*;p*βact-EGFP*	A whole BD	*D. rerio*	Dnd-KD	94.7 at prim-5 St	N/A	N/A	N/A	Con: 62.9 Dnd-KD: 50.3 TP: 26.1 at adult	N/A	[8]
*M. anguillicaudatus*	WT	50–100 BMs	*D. rerio*	WT	1.5 at 24 h	N/A	N/A	N/A	75.8 at 24 h	N/A	[13]
*O. mykiss*	WT, mid-blastula (2.5 d)	~80 BMs	*O. mykiss*	WT, early blastula (1.5 d)	N/A	31.6 at 2 y	55.6 at 2 y	10.0 at 2 y	8.0 at 2 y	0.3–14.6	[7]
*O. latipes*	TG:p*LFABP-rfp*;p*vasa-GFP*	30 BMs	*O. latipes*	WT	47.2 at St 18–23	N/A	N/A	N/A	N/A	4.3	[64]
	TG:p*LFABP-rfp*;p*vasa-GFP*, *dnd* mRNA injected			WT	81.2 at St 18–23	N/A	N/A	N/A	N/A	31.4	
	TG:p*LFABP-rfp*;p*vasa-GFP*, *dnd* mRNA injected			Dnd-KD	81.1 at St 18–23	91.4 at SM	N/A	N/A	N/A	96.5	
	TG:p*LFABP-rfp*;p*vasa-GFP*, *dnd* mRNA injected			γ-irradiated	79.2 at St 18–23	N/A	N/A	N/A	N/A	N/A	

3N: triploid, BD: blastoderm, BM: blastomere, Con: control, CP: cryopreserved, Dnd: dead end, KD: knockdown, N/A: not available, SM: sexual maturity, St: stage, TG: transgenic, TP: transplantation, WT: wild-type.

**Table A2:** Primordial germ cell transplantation in fish.

Donor				Enrichment		Recipient			% of Recipients with Gonadal Incorporation of Donor Cells	% of Recipients Producing Donor-Derived Gametes			Survival of Recipients (%)	% of Donor-Derived Offspring	Ref
Species	Fish	Age	Cells	Method	Purity	Species	Fish	Age		Total	Male	Female			
*A. japonica*	WT	Somito-genesis	1 PGC	MC	N/A	*D. rerio*	WT	Blastula	42.7 at 2 d	N/A	N/A	N/A	Con: 92.7 TP: 92.6 at 2 d	N/A	[161]
*C. auratus*	WT	10–15 somite St	1 PGC	MC	N/A	*D. rerio*	Dnd-KD	Blastula	N/A	N/A	Y at 10–12 mo	N	N/A	N/A	[16]
	WT	10–15 somite St	1 PGC	MC	N/A	*D. rerio*	Dnd-KD	Blastula	63.5 at 2 d	N/A	N/A	N/A	69.0 at 2 d	N/A	[13]
	Albino	10–15 somite St	1 PGC	MC	N/A	*C. auratus*	Dnd-KD	Blastula	42.7 at 3 d	42.9 at 5 mo–2 y	62.5 at 5 mo–2 y	30.1 at 5 mo–2 y	Dnd-KD: 88.3 TP: 84.9	92.5–100	[65]
*D. albolineatus*	WT	10–15 somite St	1 PGC	MC	N/A	*D. rerio*	Dnd-KD	Blastula	45.1 at 2 d	89.5 at SM	93.8 at SM	66.7 at SM	Dnd-KD: 59.2 TP: 74.7 at 2 d	100	[16]
	WT	10–15 somite St	1 PGC	MC	N/A	*D. rerio*	Dnd-KD	Blastula	40.2 at 2 d	N/A	N/A	N/A	76.8 at 2 d	N/A	[13]
*D. rerio*	WT	10–15 somite St	1 PGC	FACS	87.8–100	*D. rerio*	Dnd-KD	Blastula	31.5 at 1 dpt	N/A	N/A	N/A	Dnd-KD: 92.9 Dnd-KD + TP: 81.4 at 1 dpt	N/A	[17]
	WT	10–15 somite St	1 PGC	MC	N/A	*D. rerio*	Dnd-KD	Blastula	30.0 at 2 d	N/A	N/A	N/A	89.7 at 2 d	N/A	[13]
		21–25 somite St							10.1 at 2 d	N/A	N/A	N/A	82.4 at 2 d	N/A	
		Prim-5 St							5.3 at 2 d	N/A	N/A	N/A	78.4 at 2 d	N/A	
		Prim-15 St							4.5 at 2 d	N/A	N/A	N/A	87.3 at 2 d	N/A	
	WT	10–15 somite St	1 PGC	MC	N/A	*D. rerio*	Dnd-KD	Blastula	20.7 at prim-5 St	N/A	N/A	N/A	Con: 74.1 Dnd-KD: 59.4 TP: 66.7	N/A	[8]
*M. anguillicaudatus*	WT	10–15 somite St	1 PGC	MC	N/A	*D. rerio*	Dnd-KD	Blastula	N/A	N/A	Y at 10–12 mo	N	N/A	N/A	[16]
	WT	10–15 somite St	1 PGC	MC	N/A	*D. rerio*	Dnd-KD	Blastula	43.1 at 2 d	N/A	N/A	N/A	85.7 at 2 d	N/A	[13]
*O. masou*	WT	40 d	5–10 PGCs	MC	N/A	*O. mykiss*	WT	Newly hatched	24.4 at 10 dpt	N/A	N/A	N/A	N/A	N/A	[66]
*O. mykiss*	TG:p*vasa-GFP*	35 d	5–10 PGCs	MC	N/A	*O. mykiss*	WT	2.5 d	0 at 30 dpt	N/A	N/A	N/A	26 at 30 dpt	♂: 3.9 ♀: 4.2–6.1	[15]
		35 d						6 d	0 at 30 dpt	N/A	N/A	N/A	62 at 30 dpt		
		35 d						35 d	21.6 at 30 dpt	15.4 at 1–2 y	16.7 at 1 y	14.3 at 2 y	94 at 30 dpt		
		35 d						35 d	10–20 * at 30 dpt	N/A	N/A	N/A	N/A		
		40 d						35 d	10–20 * at 30 dpt	N/A	N/A	N/A	N/A		
		45 d						35 d	0–10 * at 30 dpt	N/A	N/A	N/A	N/A		
		35 d						40 d	10–20 * at 30 dpt	N/A	N/A	N/A	N/A		
		40 d						40 d	0–10 * at 30 dpt	N/A	N/A	N/A	N/A		
		45 d						40 d	0 * at 30 dpt	N/A	N/A	N/A	N/A		
		35 d						45 d	0 * at 30 dpt	N/A	N/A	N/A	N/A		
		40 d						45 d	0 * at 30 dpt	N/A	N/A	N/A	N/A		
		45 d						45 d	0 * at 30 dpt	N/A	N/A	N/A	N/A		
	TG:p*vasa-GFP*	35 d	20 PGCs	MC	N/A	*O. masou*	WT	Newly hatched	16.7 at 30 dpt	N/A	13.5 at 1 y	Y at 17 mo	N/A	♂: 0.4	[154]
	WT	32 d	5–10 PGCs	MC	N/A	*O. mykiss*	WT	Newly hatched	12.3 at 10 dpt	N/A	N/A	N/A	N/A	N/A	[66]
	TG:p*vasa-GFP*	30 dpf	15–20 PGCs	MC	N/A	*O. mykiss*	WT	32–34 dpf	Fresh: 12.5 CP (d1): 10.1 at 30 dpt	Fresh: 10.9 CP (d1): 8.3 at 3 y	Fresh: 10.3 CP (d1): 7.8 at 3 y	Fresh: 12.1 CP (d1): 9.1 at 3 y	Fresh: 98.5 CP (d1): 86.5 at 30 dpt	Fresh ♂: 0.2–16.3 Fresh ♀: 0.2–7.6 CP (d1) ♂: 2.0–13.5 CP (d1) ♀: 0.1–3.3	[53]
*S. trutta*	WT	40 d	5–10 PGCs	MC	N/A	*O. mykiss*	WT	Newly hatched	9.4 at 10 dpt	N/A	N/A	N/A	N/A	N/A	[66]
Hybrid	*H. huso* × *A. ruthenus*	Late-neural St	1 PGC	MC	N/A	*C. auratus*	WT	Blastula	9.1 at 3 d	N/A	N/A	N/A	95.7 at 3 d	N/A	[162]

Asterisk: estimated average value from the related graphs (no numerics presented), Con: control, CP: cryopreserved, Dnd: dead end, dpt: day post-transplantation, FACS: fluorescence-activated cell sorting, KD: knockdown, MC: manually collected under a fluorescence microscope, N: no (failed, no numerical data presented), N/A: not available, PGC: primordial germ cell, SM: sexual maturity, St: stage, TG: transgenic, TP: transplantation, Y: yes (succeeded, no numerical data presented), WT: wild-type.

**Table A3:** Gonadal germ cell transplantation into the peritoneal cavity of larvae in fish.

Donor				Enrichment		Recipient			% of Recipients with Gonadal Incorporation of Donor Cells	% of Recipients Producing Donor-Derived Gametes			Survival of Recipients (%)	% of Donor-Derived Offspring	Ref
Species	Fish	Age (Size)	Cells	Method	Purity	Species	Fish	Age (Size)		Total	Male	Female			
*A. baerii*	WT	4 y	TCs	PDGC	79.4	*A. ruthenus*	WT	1 wph	60.0 at 90 dpt	N/A	N/A	N/A	Con: 96.7 TP: 95.8	N/A	[39]
	WT	4 y	TCs	N/C	N/A	*A. ruthenus*	WT	Newly hatched	Fresh: 55.0 CP: 65.0 at 90 dpt	N/A	N/A	N/A	N/A	N/A	[195]
	WT	4 y	OCs	PDGC	70.8	*A. ruthenus*	WT	1 wph	60.0 at 90 dpt	N/A	N/A	N/A	Con: 96.7 TP: 95.8	N/A	[39]
	WT	4 y	OCs	N/C	N/A	*A. ruthenus*	WT	Newly hatched	Fresh: 70.0 CP: 55.0 at 90 dpt	N/A	N/A	N/A	N/A	N/A	[195]
*A. dabryanus*	WT	2 y	50,000 TCs	N/C	N/A	*A. dabryanus*	WT	7–8 dph	70.0 at 51 dpt	N/A	N/A	N/A	Con: 86.2 TP: 76.7 at 51 dpt	N/A	[196]
*A. sinensis*	WT	11.5 y	50,000 OCs	N/C	N/A	*A. dabryanus*	WT	7–8 dph	20.0 at 51 dpt	N/A	N/A	N/A	Con: 86.2 TP: 68.6 at 51 dpt	N/A	[196]
*C. carpio*	WT	2 y	30,000–50,000 TCs	PDGC	N/A	*C. auratus*	Dnd-KD	7 d	62.5 at 1 mo	43.7 at 3 y	32.4 at 3 y	11.2 at 3 y	Con: 74 Dnd-KD: 79 Dnd-KD + TP: 72 at 18 mpf	100	[29]
*D. rerio*	TG:p*piwil1-DsRed*	2–3 mo	10–60 SG	PDGC	N/A	*D. rerio*	Dnd-KD	2 w	N/A	N/A	Cult (3 w): 19 Cult (6 w): 18 at SM	N/A	Cult (3 w): 72.2 Cult (6 w): 64.9 at SM	100	[126]
	TG:p*vasa-EGFP*	Adult	3000 GFP+ TCs	N/C	N/A	*D. rerio*	WT	9–10 d	6.7 at 50 dpt	N/A	6.7 at SM	N/A	N/A	3.9–7.3	[101]
							*Dnd*-KO		5.0 at 50 dpt	N/A	5.0 at SM	N/A	N/A	100	
	TG:p*vasa-EGFP*	2 mo	3000–5000 TCs	N/C	N/A	*D. rerio*	3N	7 d	53.2 at adult	N/A	43.5 at adult	N/A	2N Con: 83.3 3N Con: 75.6 3N TP: 68.9 at adult	100	[145]
	TG:p*vasa-EGFP* or TG:p*βact-YFP*	3–6 mo	3000 GFP+ TCs	N/C	N/A	*D. rerio*	WT	7 d	Fresh: 31.1 CP: 24.4 VF: 22.2 at 50 dpt	N/A	N/A	N/A	Contro: 80 TP: 85 at 50 dpt	N/A	[135]
							Dnd-KD		Fresh:58.3 CP: 47.4 VF: 50.0 at 50 dpt	N/A	43 at 6 mo	N/A	N/A	100	
	TG:p*vasa-DsRed2*- *vasa*;p*βact-EGFP*	3 mo	OCs	PDGC	N/A	Hybrid	♂ *D. albolineatus* × ♀ *D. rerio*	2 w	20.0 at 8 w	17.9 at 6 mo	17.9 at 6 mo	0 at 6 mo	52.7 at 6 mo	100	[69]
	TG:p*ziwi-Neo*;p*ziwi-DsRed*	10–12 w	20–40 FGSCs	PDGC	N/A	*D. rerio*	Dnd-KD	2 w	N/A	N/A	Cult (3 w): 20.0 Cult (6 w): 16.0 at SM	N/A	Cult (3 w): 73.5 Cult (6 w): 71.7 at SM	100	[123]
	TG:p*vasa-EGFP*	2 mo	500 OCs	N/C	N/A	*D. rerio*	3N	7 d	43.6 at adult	N/A	34.5 at adult	N/A	2N Con: 83.3 3N Con: 75.6 3N TP: 61.1 at adult	100	[145]
*N. mitsukurii*	WT	3 mo	10,000 TCs	N/C	N/A	*S. japonicus*	WT	5 d	33.5 at 21 dpt	N/A	N/A	N/A	20.3 at 21 dpt	N/A	[99]
								7 d	70.0 at 21 dpt	N/A	N/A	N/A	20.6 at 21 dpt	N/A	
								9 d	4.4 at 21 dpt	N/A	N/A	N/A	27.6 at 21 dpt	N/A	
	WT	3 mo	3000 TCs	FACS	Unsort: 18.8 Sort: 94.0	*N. mitsukurii*	WT	12 d	Unsort: 7.6 Sort: 60.0 at 20 dpt	N/A	N/A	N/A	N/A	N/A	[19]
	TG:p*HSC-GFP*	3 mo	10,000 TCs	N/C	N/A	*N. mitsukurii*	3N (<100% incidence)	12 dph	2N: 63.3 3N: 56.0 at 18 dpt	2N: 6.1 3N: 31.4 at 6 mo	2N: 5.3 3N: 36.8 at 6 mo	2N: 6.8 3N: 28.9 at 6 mo	41.1 at 18 dpt	2N ♂: 6.8–89.2 3N ♂: 100 2N ♀: 2.2–25.0 3N ♀: 100	[63]
	TG:p*HSC-GFP*	3 mo	10,000 TCs	N/C	N/A	*N. mitsukurii*	WT	12 d	58.3 at 2 wpt	N/A	N/A	N/A	68.1 at 2 wpt	N/A	[27]
						Hybrid	♂ *P. argentata* × ♀ *N. mitsukurii*, 3N	12 d	58.6 at 2 wpt	34.4 at 6 mo	34.4 at 6 mo	0 at 6 mo	34.7 at 2 wpt	♂: 100	
*O. masou*	WT	12–13 mo	3000 TCs	FACS	Unsort: 16.4 Sort: 75.6	*O. mykiss*	WT	Newly hatched	Unsort: 0–25 * Sort: 80.0 at 20 dpt	N/A	N/A	N/A	N/A	N/A	[19]
	WT, black	8 mo	50,000 TCs	N/C	N/A	*O. masou*	3N	40–42 d	Fresh: 50–60 * CP: 58.2 at 50 dpt	CP: 39.1 at 2 y	CP: 43.5 at 2 y	CP: 34.8 at 2 y	N/A	100	[28]
	WT, black	8 mo	50,000 OCs	N/C	N/A	*O. masou*	3N	40–42 d	Fresh: 40–60 * CP: 41.2 at 50 dpt	CP: 25.8 at 2 y	CP: 29.3 at 2 y	CP: 23.1 at 2 y	N/A	100	[28]
*O. mykiss*	Albino, TG:p*vasa-GFP*	9 mo	18,000 TCs (10,000 GFP+ cells)	FACS	N/A	*O. mykiss*	WT	Newly hatched	♂: 38.8 ♀: 57.8 at 2 mpt	N/A	50 at 1 y	40 at 2 y	N/A	♂: 5.5 (0.2–40.5) ♀: 2.1 (0.1–9.9)	[37]
	TG:p*vasa-GFP*	Adult	TCs	N/C	N/A	*O. masou*	3N	Newly hatched	N/A	N/A	34.5 at 2 y	50.0 at 17 mo; 10.0 at 2–3 y	N/A	♂: 100 ♀: 100	[146]
	TG:p*vasa-GFP*	8–10 mo	10,000 TCs	DP	95–99	*O. mykiss*	WT	Newly hatched	Sort: 77.7 Sort + Cult (1 mo): 52.2 at 30 dpt	N/A	N/A	N/A	N/A	N/A	[75]
	TG:p*vasa-GFP*	16 mo	3000 TCs	FACS	N/A	*O. mykiss*	WT	Newly hatched	Sort-A: 51.0 Sort-B: 0 at 60 dpt	N/A	N/A	N/A	N/A	N/A	[67]
		7 mo	3000 ASG	N/C	N/A				53.6 at 60 dpt	N/A	N/A	N/A	N/A	N/A	
	WT	8–12 mo	3000 TCs	FACS	N/A	*O. mykiss*	WT	Newly hatched	Unsort: 23.0 Sort: 78.3 at 20 dpt	N/A	N/A	N/A	N/A	N/A	[19]
	TG:p*vasa-GFP*	8–12 mo			Unsort: 35.9 Sort: 93.2				Unsort: 29.0 Sort: 82.0 at 20 dpt	N/A	N/A	N/A	N/A	N/A	
	TG:p*vasa-GFP*	16 or 24 mo			Sort: 90.0–96.2				N/A	N/A	N/A	N/A	N/A	N/A	
	TG:p*vasa-GFP*	11–12 mo	5000 TCs	DP	>90	*O. mykiss*	WT	25–27 d	Fresh: 59.7 Cult + Sort: 94.5 Cult + Sort + Tryp-2 h: 43.9 at 20 dpt	N/A	N/A	N/A	N/A	N/A	[73]
	TG:p*Mlc2-GFP*	6 mo	10,000 ASG	PDGC + CE	>90	*O. mykiss*	WT	Peri-hatching	90 at 6 mo	N/A	90 at 1–2 y	N/A	N/A	1–56	[23]
		1 y			>90				78 at 6 mo	N/A	N/A	N/A	N/A	N/A	
		Mature			>70				42 at 6 mo	N/A	N/A	N/A	N/A	N/A	
	TG:p*vasa-GFP*	11 mo	500 GFP+ TCs	N/C	N/A	*O. mykiss*	3N	32 d	Fresh: 30–40 * CP (F371): 30–40 * at 50 dpt	Fresh: 32.4 CP (F371): 28.6 at 2 y	Fresh: 35.0 CP (F371): 40.0 at 2 y	Fresh: 29.4 CP (F371): 15.4 at 2 y	N/A	100	[61]
		18 mo				*O. mykiss*		32 d	Fresh: 40–60 * CP (F371): 40–60 * at 30 dpt	N/A	N/A	N/A	N/A	N/A	
		11 mo				*O. masou*		37 d	Fresh: 20–30 * CP (F371): 20–30 * at 50 dpt	Fresh: 27.6 CP (F371): 24.0 at 2 y	Fresh: 33.3 CP (F371): 30.8 at 2 y	Fresh: 23.5 CP (F371): 16.7 at 2 y	N/A	100	
	TG:p*vasa-GFP*	8 mo	30,000 TCs	N/C	N/A	*O. masou*	WT	38–40 d	63.2 at 20 dpt	N/A	N/A	N/A	N/A	N/A	[124]
							Dnd-KD		70 at 20 dpt	54.5 at 2 y	46.7 at 2 y	71.4 at 2 y	N/A	♂: 100 (6 Inds); 72.3 (1 Ind) ♀: 100 (2 Inds); 21.1 (3 Inds)	
	TG:p*vasa-GFP*	9–11 mo	1000 TCs	FACS (AB No. 80 or No. 95)	Unsort: <20 * Sort (No. 80): 70.7 Sort (No. 95): 80.9	*O. mykiss*	WT	Newly hatched	Unsort: 1.1 Sort (No. 80): 19.2 Sort (No. 95): 18.4 at 20 dpt	N/A	N/A	N/A	N/A	N/A	[20]
	TG:p*vasa-GFP*	13–14 mo	<2000 TCs	MACS (AB No. 172)	Unsort: 49.8 Sort: 81.7	*O. mykiss*	WT	Newly hatched	Unsort: 10–20 * Sort: 30–40 * at 20 dpt	N/A	N/A	N/A	N/A	N/A	[62]
	TG:p*vasa-GFP*	12–15 mo	15,000 SG	DP	N/A	*O. mykiss*	3N	30 d	Fresh: 60–80 * Cult: 60–80 * at 20 dpt	Fresh: 89.2 Cult: 65.0 at 1 y	Fresh: 88.0 Cult: 77.6 at 1 y	Fresh: 73.3 Cult: 36.8 at 2 y	N/A	♂: 100 ♀: 100	[147]
	TG:p*vasa-GFP*	6–9 mo	15,000 OCs	N/C	N/A	*O. mykiss*	3N	25 d	♂: 50.0 ♀: 47.1 at 5 mo	10.0 at 2 y	15.0 at 2 y	5.0 at 2 y	N/A	♂: 100 ♀: 100	[21]
	TG:p*vasa-GFP*	9 mo	<10,000 GPF+ OCs	N/C	N/A	*O. mykiss*	3N	33 d	Fresh: 60–80 * CP: 57.1 at 50 dpt	Fresh: 25.0 CP: 24.0 at 2.5 y	Fresh: 30.8 CP: 28.0 at 2.5 y	Fresh: 18.2 CP: 20.0 at 2.5 y	N/A	Fresh: 100 CP: 100	[56]
	TG:p*vasa-GFP*	12–14 mo	<10,000 OCs	MACS (AB No. 172)	Unsort: 5.2 Sort: 54.8	*O. mykiss*	WT	Newly hatched	Unsort: 0–20 * Sort: 60–80 * at 20 dpt	N/A	N/A	N/A	N/A	N/A	[62]
*O. nerka*	WT	15 mo	<5000 TCs	MACS (AB No. 172)	Unsort: 16.4 Sort: 68.6	*O. mykiss*	WT	Newly hatched	Unsort: 0–20 * Sort: 40–60 * at 20 dpt	N/A	N/A	N/A	N/A	N/A	[62]
*O. niloticus*	TG:p*βact-EGFP*	5–12 mo	20,000 TCs	N/C	N/A	*O. niloticus*	WT	6 d	0 at 5 mo	N/A	N/A	N/A	N/A	N/A	[137]
								7 d	6.7 at 5 mo	37.5 at 1 y	40.0 at 1 y	33.3 at 1 y	N/A	2.4	
								8 d	0 at 5 mo	N/A	N/A	N/A	N/A	N/A	
*O. latipes*	TG:p*vasa-GFP*	2–4 mo	15,000 TCs	N/C	N/A	*O. latipes*	WT	7 d	>50 * at 1 mpt	N/A	N/A	N/A	20.0 at 1 mpt	N/A	[55]
			15,000 TCs				WT	11 d	>50 * at 1 mpt	N/A	N/A	N/A	90 at 1 mpt	N/A	
			15,000 TCs				WT	14 d	<50 * at 1 mpt	N/A	N/A	N/A	90 at 1 mpt	N/A	
			15,000 TCs				WT	19 d	0 * at 1 mpt	N/A	N/A	N/A	90 at 1 mpt	N/A	
			15,000 TCs				WT	23 d	0 * at 1 mpt	N/A	N/A	N/A	90 at 1 mpt	N/A	
			300 TCs				WT	11 d	10 at 1 mpt	N/A	N/A	N/A	N/A	N/A	
			1000 TCs				WT	11 d	10 at 1 mpt	N/A	N/A	N/A	N/A	N/A	
			3000 TCs				WT	11 d	60 at 1 mpt	N/A	N/A	N/A	N/A	N/A	
			10,000 TCs				WT	11 d	60 at 1 mpt	N/A	N/A	N/A	N/A	N/A	
			30,000 TCs				WT	11 d	60 at 1 mpt	N/A	N/A	N/A	N/A	N/A	
			15,000 TCs				3N	11 d	N/A	Fresh: 63.6 CP: 70.0 at 2–3 mo	N/A	N/A	N/A	100	
	*Wnt4b* (-/-) mutant	-	15,000 TCs	N/C	N/A	*O. latipes*	3N	11 d	N/A	CP: 83.3 at 2–3 mo	N/A	N/A	N/A	100	[55]
	Tokyo-medaka (endangered)	1 y					TG:p*vasa-GFP*, 3N		N/A	CP: 63.9 at 2–3 mo	N/A	N/A	N/A	100	
	Kaga (inbred)	-					TG:p*vasa-GFP*, 3N		N/A	CP: 58.6 at 2–3 mo	N/A	N/A	N/A	100	
	*Fshr* mutant (-/-), XX male	3 mo	3000–9000 TCs	N/C	N/A	Hybrid	♂ *O. curvinotus* × ♀ *O. latipes* or ♂ *O. latipes* × ♀ *O. curvinotus*	Newly hatched	N/A	N/A	N/A	35.3 at 3 mo	N/A	100	[144]
	TG:p*vasa-GFP*	2 mo	12,000 TCs						N/A	N/A	N/A	48.3 at 3 mo	N/A	N/A	
	WT	Adult	1000 OCs	PDGC + DP	N/A	*O. latipes*	WT	11 d	[Cult (10 d)] Con: 15.0 On pDA: 25.0 On pDA/PLL: 0 at 20 d	N/A	N/A	N/A	50.0–80.0 at 20 d	N/A	[197]
	WT	Adult	1000 OCs	PDGC + DP	N/A	*O. latipes*	WT	11 d	Con: 14.3 Sort: 32.1 at 20 d	N/A	N/A	N/A	57.1–72.4 at 20 d	N/A	[70]
*P. dentatus*	WT	N/A	TCs	PDGC	N/A	*P. olivaceus*	3N	17 dph	100 at 14 dpt	N/A	Merged: 100 at 2 y	N/A	[Merged] Con: 27.1 at 3 y TP: 4.0 at 2 y	Merged: Y	[148]
								20 dph	90.0 at 14 dpt	N/A		N/A			
*P. olivaceus*	WT	N/A	TCs	PDGC	N/A	*P. olivaceus*	3N	16 dph	100 at 14 dpt	N/A	Merged: 100 at 2 y	N/A	[Merged] Con: 27.1 at 3 y TP: 6.8 at 2 y	Merged: Y	[148]
								19 dph	100 at 14 dpt	N/A		N/A			
								22 dph	100 at 14 dpt	N/A		N/A			
*P. spathula*	WT	2.5 y	50,000 TCs	PDGC	N/A	*A. dabryanus*	WT	7–8 dph	80.6 at 2 mo	N/A	N/A	N/A	Con: 76.5 TP: 68.5 at 2 mo	N/A	[198]
*S. salar*	WT	1 y	2000–5000 TCs	N/C	N/A	*O. mykiss*	3N (Shasta/ Shasta/albino)	Newly hatched	61.1 at 4 wpt	11.0 at 1–2 y	10.0 at 1–2 y	12.1 at 2 y	N/A	♂: 100 ♀: N/A	[150]
*S. trutta*	WT	9–12 mo	4000–5000 TCs	FACS (AB No. 95)	Unsort: 21.2 Sort: 80.6	*O. mykiss*	WT	Newly hatched	Con: 15.8 Sort: 34.7 at 20 dpt	N/A	N/A	N/A	N/A	N/A	[86]
*S. leucomaenis*	WT	12–13 mo	3000 TCs	FACS	Unsort: 10.1 Sort: 79.9	*O. mykiss*	WT	Newly hatched	Unsort: 0 Sort: 48.8 at 20 dpt	N/A	N/A	N/A	N/A	N/A	[19]
*S. maximus*	WT	N/A	TCs	PDGC	N/A	*P. olivaceus*	3N	15 dph	60.0 at 14 dpt	N/A	Merged: 40.0 at 2 y	N/A	[Merged] Con: 27.1 at 3 y TP: 10.0 at 2 y	Merged: Y	[148]
								18 dph	40.0 at 14 dpt	N/A		N/A			
								21 dph	30.0 at 14 dpt	N/A		N/A			
*S. quinqueradiata*	WT	10 mo	20,000 TCs	N/C	N/A	*S. quinqueradiata*	WT	6 dph	100 at 28 dpt	N/A	N/A	N/A	8.0 at 28 dpt	N/A	[155]
								8 dph	100 at 28 dpt	N/A	N/A	N/A	10.9 at 28 dpt	N/A	
								10 dph	85.7 at 28 dpt	N/A	N/A	N/A	15.9 at 28 dpt	N/A	
								12 dph	85.7 at 28 dpt	N/A	N/A	N/A	29.6 at 28 dpt	N/A	
								Merged	N/A	N/A	100 at 1.5–2.5 y	100 at 2.5 y	5.08 at SM	♂: 66.6 (19.6–98.8) ♀: 63.2 (17.0–97.5)	
*T. rubripes*	WT	1 y	4000–6000 TCs	N/C	N/A	*T. alboplumbeus*	3N (<100% incidence)	1 dph	50 at 4 wpt	N/A	38.3 at 11 mo	31.3 at 2 y	47 at 72 wpt	♂: 100 ♀: 5–95	[138]
								5 dph	33.3 at 4 wpt	N/A	23.7 at 11 mo	23.1 at 2 y	41.4 at 72 wpt		
	WT	10–12 mo	5000 TCs	N/C	N/A	*T. alboplumbeus*	3N (<100% incidence)	1 dph	Fresh: 39.1 CP: 38.9 at 40 dph	N/A	[CP] 2N: 36.4 3N: 64.2 at 10 mph	[CP] 2N: 0 3N: 1.6 at 2 y	Con: 41.4 Fresh: 21.8 CP: 14.5 at 40 dph	2N ♂: 3.2–87.5 (3 Inds) 2N ♀: 0 (10 Inds) 3N ♂: 100 (11 Inds); 62.5 (1 Ind) 3N ♀: 100 (1 Ind)	[120]
	WT	1 y	5000 TCs	N/C	N/A	*T. alboplumbeus*	WT	1 dph	40.0 at 40 dph	N/A	N/A	N/A	Con: 23.8 at 40 dph; 45.3 at 12 mph TP: 14.8 at 40 dph	N/A	[127]
							Dnd-KD		40.5 at 40 dph	N/A	91.7 at 10 mo	26.7 at 2 y	Dnd-KD: 20.3 and 35.0 Dnd-KD + TP: 15.8 and 16.9 at 40 dph and 12 mph	♂: 100 (7 Inds); 12.5–87.5 (2 Inds) ♀: 100 (3 Inds)	
*T. maccoyii*	WT	57.0 kg	TCs	PDGC	N/A	*S. lalandi*	WT	6–7 dph	33.3 at 18 dpt	N/A	N/A	N/A	9.1 at 64 dph	N/A	[47]
								9–10 dph	37.5 at 18 dpt	N/A	N/A	N/A	9.2 at 64 dph	N/A	
*T. orientalis*	WT	0 y	TCs	N/C	N/A	*S. japonicus*	WT	7 d	0 at 21 dpt	N/A	N/A	N/A	N/A	N/A	[22]
		1 y							5.4 at 21 dpt	N/A	N/A	N/A	N/A	N/A	
		3 y							0 at 21 dpt	N/A	N/A	N/A	N/A	N/A	
	WT	2–4 y	>10,000 TCs	FACS (AB No. 152)	Unsort: 16.6 Sort: 77.3	*N. mitsukurii*	WT	12 dph	PDGC Sort (PKH26 labled): 33.3 PDGC Sort (No. 152 AB labeled): 63.3 at 14 dpt	N/A	N/A	N/A	N/A	N/A	[45]
	WT	3 y	>10,000 TCs	PDGC	N/A	Hybrid	♂ *S. japonicus* × ♀ *S. australasicus*	10 d	100 at 14 dpt	N/A	N/A	N/A	N/A	N/A	[48]
	WT	2 y	>30,000 TCs	N/C	N/A	*S. japonicus*	WT	7 dph	Y at 14 dpt and 4 mpt	N/A	N/A	N/A	N/A	N/A	[46]
						*E. affinis*	WT	10 dph	Y at 14 dpt and 3 mpt	N/A	N/A	N/A	N/A	N/A	

2N: diploid, 3N: triploid, AB: antibody, ASG: type A spermatogonia, Asterisk: estimated average value from the related graphs (no numerics presented), CE: centrifugal elutriation, Con: control, CP: cryopreserved, Cult: cultured, Dnd: dead end, DP: differential plating, dph: day post-hatching, dpt: day post-transplantation, FACS: fluorescence-activated cell sorting, FGSC: female germline stem cell, Ind: individual, KD: knockdown, KO: knockout, MACS: magnetic-activated cell sorting, mpt: month post-transplantation, N/A: not available, N/C: not carried out, OC: ovarian cell, PDGC: Percoll density gradient centrifugation, SG: spermatogonia, SM: sexual maturity, Sort: sorted, TC: testicular cell, TG: transgenic, TP: transplantation, Trip: trypsinized, Unsort: unsorted, VF: vitrified, wph: week post-hatching, wpt: week post-transplantation, WT: wild-type, Y: yes (succeed, no numerical data presented).

**Table A4:** Gonadal germ cell transplantation into adult recipients in fish.

Donor				Enrichment		Recipient				% of Recipients with Gonadal Incorporation of Donor Cells	% of Recipients Producing Donor-Derived Gametes			Survival of Recipients (%)	% of Donor-Derived Offspring	Ref
Species	Fish	Age (Size)	Cells	Method	Purity	Species	Fish	Sex	Age (Size)		Total	Male	Female			
*D. rerio*	TG:p*vasa* -*EGFP*	Adult	TCs	FACS	Unsort: 10–20 * Sort: 60–70 *	*D. rerio*	WT, HT + B treated	♂	Adult	30 at 3 wpt	N/A	N/A	N/A	N/A	N/A	[128]
								♀	Adult	Y at 3 wpt	N/A	N/A	N/A	N/A	N/A	
*I. furcatus*	WT	2 y	2 × 10^4^–1.43 × 10^6^ TCs	PDGC	N/A	*I. punctatus*	3N	♂	2 y	87.5 at 10 mpt	N/A	Y at 2 ypt	N/A	N/A	Y	[199]
*N. mitsukurii*	TG:p*HSC* -*GFP*	3 mo	2 × 10^6^ TCs	N/C	N/A	Hybrid	♂ *P. argentata* × ♀ *N. mitsukurii*	♂	6 mo	37.5 at 6 wpt	N/A	10 at 25 wpt	N/A	77.0 at 1 dpt	100	[31]
	TG:p*HSC* -*GFP*	3 mo	2 × 10^6^ OCs	N/C	N/A	Hybrid	♂ *P. argentata* × ♀ *N. mitsukurii*, 3N	♀	6 mo	N/A	33.3 at 9 mpt	16.6 at 9 mpt	16.6 at 9 mpt	75 at mpt	100	[104]
*O. bonariensis*	WT	4–5 mo	4 × 10^4^ TCs (for each lobe, surgery)	PDGC	N/A	*O. hatcheri*	WT, HT + B treated	♂	1 y	33.3 at 6 wpt	N/A	20.0 at 6 mpt	N/A	N/A	1.2–13.3	[149]
	WT	4–5 mo	3.75 × 10^6^ TCs	PDGC	N/A	*O. hatcheri*	WT, HT + B treated	♂	1 y	80.0 at 8 wpt	N/A	17.6 at 7 mpt	N/A	N/A	12.6–39.7	[100]
	WT	4–5 mo	3.75 × 10^6^ Ocs	PDGC	N/A	*O. hatcheri*	WT, HT + B treated	♀	1 y	60.0 at 8 wpt	N/A	N/A	5.0 at 7 mpt	N/A	39.7–52.2	[100]
*O. niloticus*	WT	Adult	1 × 10^7^ TCs	PDGC + DP	N/A	*O. niloticus*	WT, HT + B treated	♂	Adult	89.5 at 8–9 wpt	N/A	Y at 9 wpt	N/A	N/A	6.3	[32]
*P. olivaceus*	WT	1 y	OCs	PDGC	Unsort: 37.8 Sort: 83.6	*P. olivaceus*	WT, HT + B treated	♂	2 y	Y at 123 dpt	N/A	11.7 at 10 mpt	N/A	100 at 10 mpt	43.0–45.5	[72]
*R. quelen*	WT	289 g	1 × 10^7^ TCs	PDGC + DP	N/A	*O. niloticus*	WT, HT + B treated	♂	208 g	100 at 2 mpt	N/A	N/A	N/A	N/A	N/A	[163]

3N: triploid, Asterisk: estimated average value from the related graphs (no numerics presented), B: busulfan, DP: differential plating, dpt: day post-transplantation, FACS: fluorescence-activated cell sorting, HT: high temperature, mpt: month post-transplantation, N/A: not available, N/C: not carried out, OC: ovarian cell, PDGC: Percoll density gradient centrifugation, Sort: sorted, TC: testicular cell, Unsort: unsorted, wpt: week post-transplantation, WT: wild-type, Y: yes (succeed, no numerical data presented), ypt: year post-transplantation.
